# Supplementary material for: SIX1 and EWS/FLI1 co-regulate an anti-metastatic gene network in Ewing Sarcoma
Source: Nat Commun. 2023 Jul 19;14:4357. doi: 10.1038/s41467-023-39945-w (PMC10356808; doi:10.1038/s41467-023-39945-w)

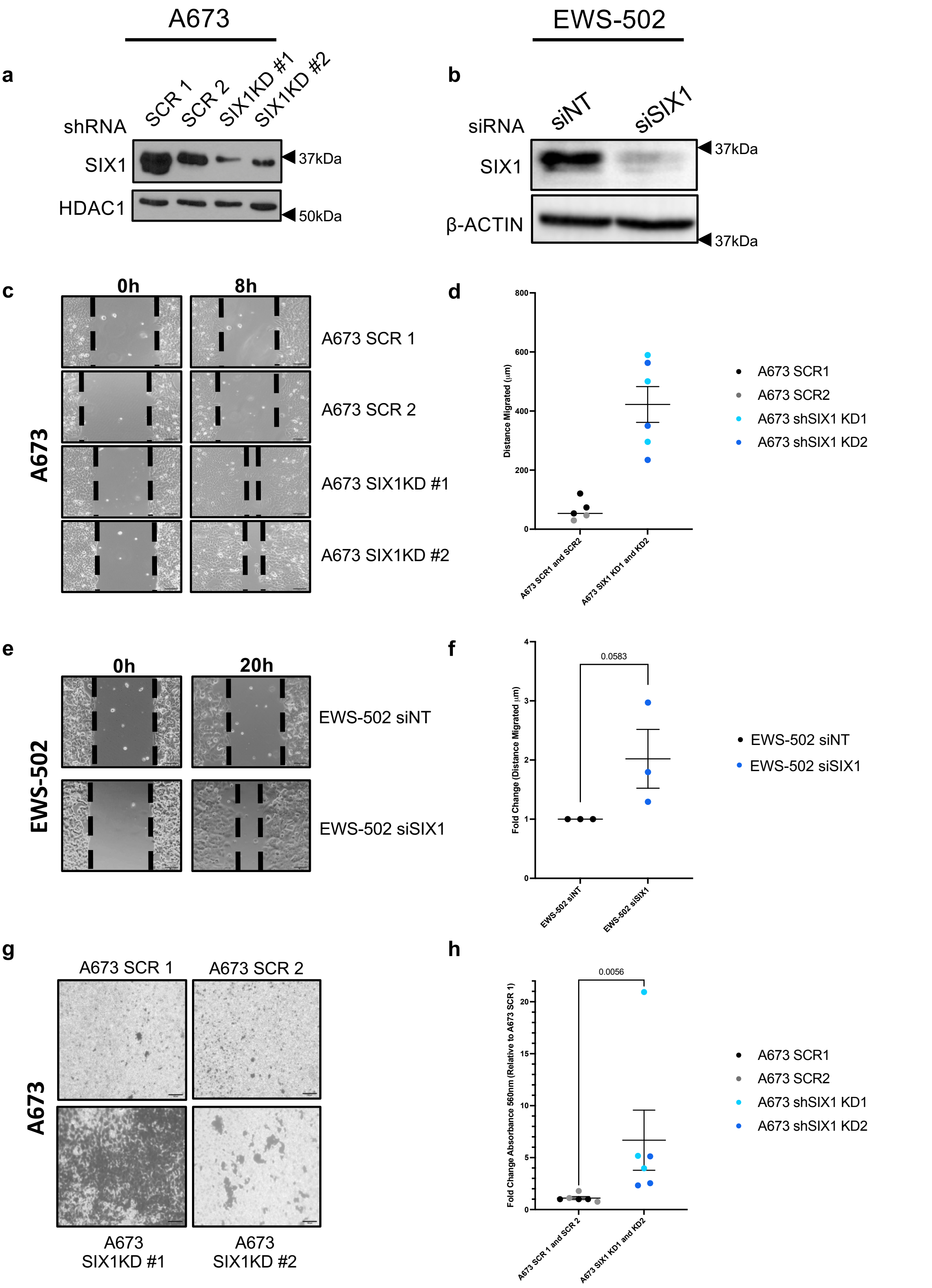

Supplementary Figure 1. Alternative methods of SIX1 KD confirm an anti-migratory and anti-invasive effect for SIX1. (a) Representative Western blot analysis displaying SIX1 levels in clonal SCR or SIX1 KD cells in the A673 cell line. (b) Representative Western blot analysis displaying SIX1 levels in EWS-502 cells treated with either non-targeting (siNT) or siSIX1 siRNAs. (c) Representative scratch wound migration assay comparing A673 clonal SCR or SIX1 KD cells at 0h and 8h after plating. (d) Quantification of scratch wound migration assays. Mean±SEM of n=3 independent experimental replicates of A673 SCR1, shSIX1 KD1 and shSIX1 KD2 (n=2 experimental replicates of A673 SCR 2). Each experimental replicate consists of n=3 technical replicates (averaged). (e) Representative scratch wound migration assay comparing EWS-502 siNT an siSIX1 cells at 0h and 20h after plating. (f) Quantification of scratch wound migration assays (fold change distance migrated relative to EWS-502 siNT). Mean±SEM of n=3 independent experimental replicates shown. Each experimental replicate consists of n=3 technical replicates (averaged). (g) Representative images of transwell invasion inserts comparing invasion of clonal A673 SCR or SIX1 KD cells (h) Quantification of transwell invasion assays (fold change absorbance 560nm relative to A673 SCR 1). Mean±SEM of n=3 independent experimental replicates of A673 SCR1, SCR2, shSIX1 KD1, and shSIX1 KD2 (SCR and SIX1 KD replicates combined for analysis). Each experimental replicate consists of n=3 technical replicates (averaged). Statistical analysis for f. and h. performed using one-tailed ratio T-test. Uncropped Western blot images can be found at the end of the Supplementary File. Source data are available as a Source Data file.

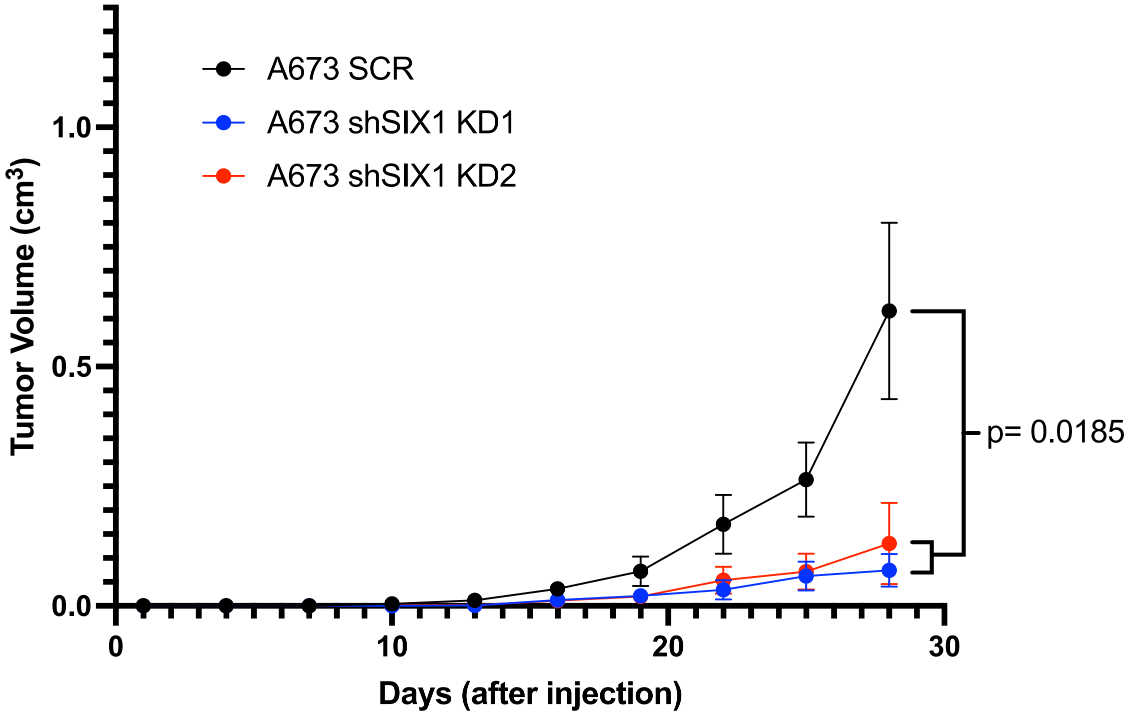

Supplementary Figure 2. SIX1 KD attenuates subcutaneous tumor growth. Caliper measurements of A673 SCR or SIX1 KD tumors after subcutaneous flank injection in NSG mice. A673 SCR (n=6 mice), shSIX1 KD1 (n=5 mice), and shSIX1 KD2 (n=4 mice, one mouse excluded from after being determined to be a significant outlier by the Grubbs test). Mean±SEM shown. Statistical analysis performed by fitting data to a longitudinal mixed-effects model. Source data are available as a Source Data file.

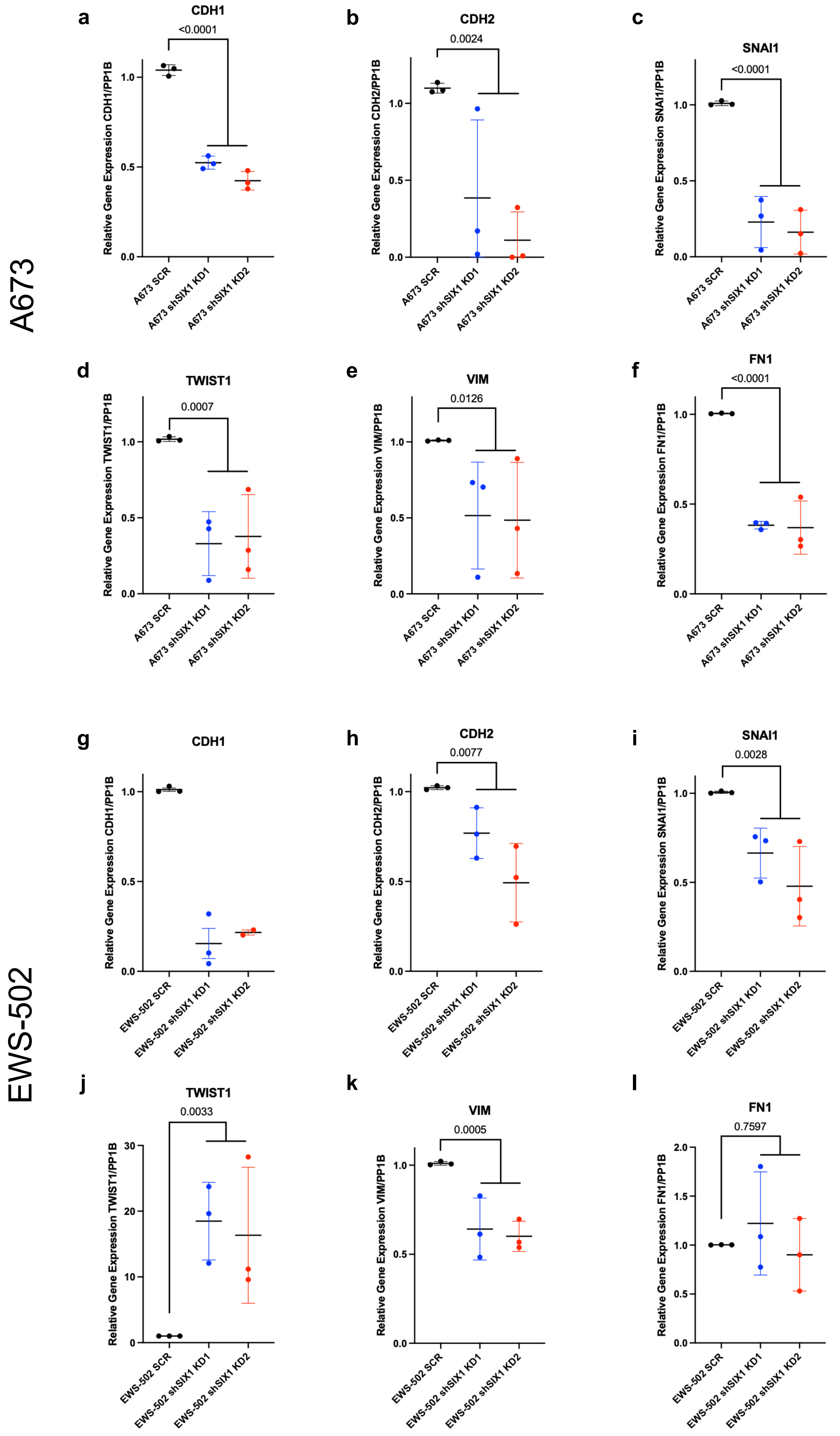

Supplementary Figure 3. SIX1 KD leads to mixed EMT transcriptional signature in A673 and EWS-502 cells. Gene expression as measured by qRT-PCR of (a) CDH1, (b) CDH2, (c) SNAI1, (d) TWIST1, (e) VIM, and (f) FN1 between A673 SCR and shSIX1 KD cells. Gene expression of (g) CDH1, (h) CDH2, (i) SNAI1, (j) TWIST1, (k) VIM, and (l) FN1 between EWS-502 SCR and shSIX1 KD cells. Mean±SEM of n=3 independent experimental replicates shown (n=2 for EWS-502 shSIX1 KD2 CDH1 due to exclusion of a significant outlier as determined by Grubbs' test). Each experimental replicate consists of n=3 technical replicates (averaged). Statistical analysis comparing SCR to combined SIX1 KD replicates performed using unpaired two-tailed Welch's T-test. Source data are available as a Source Data file.

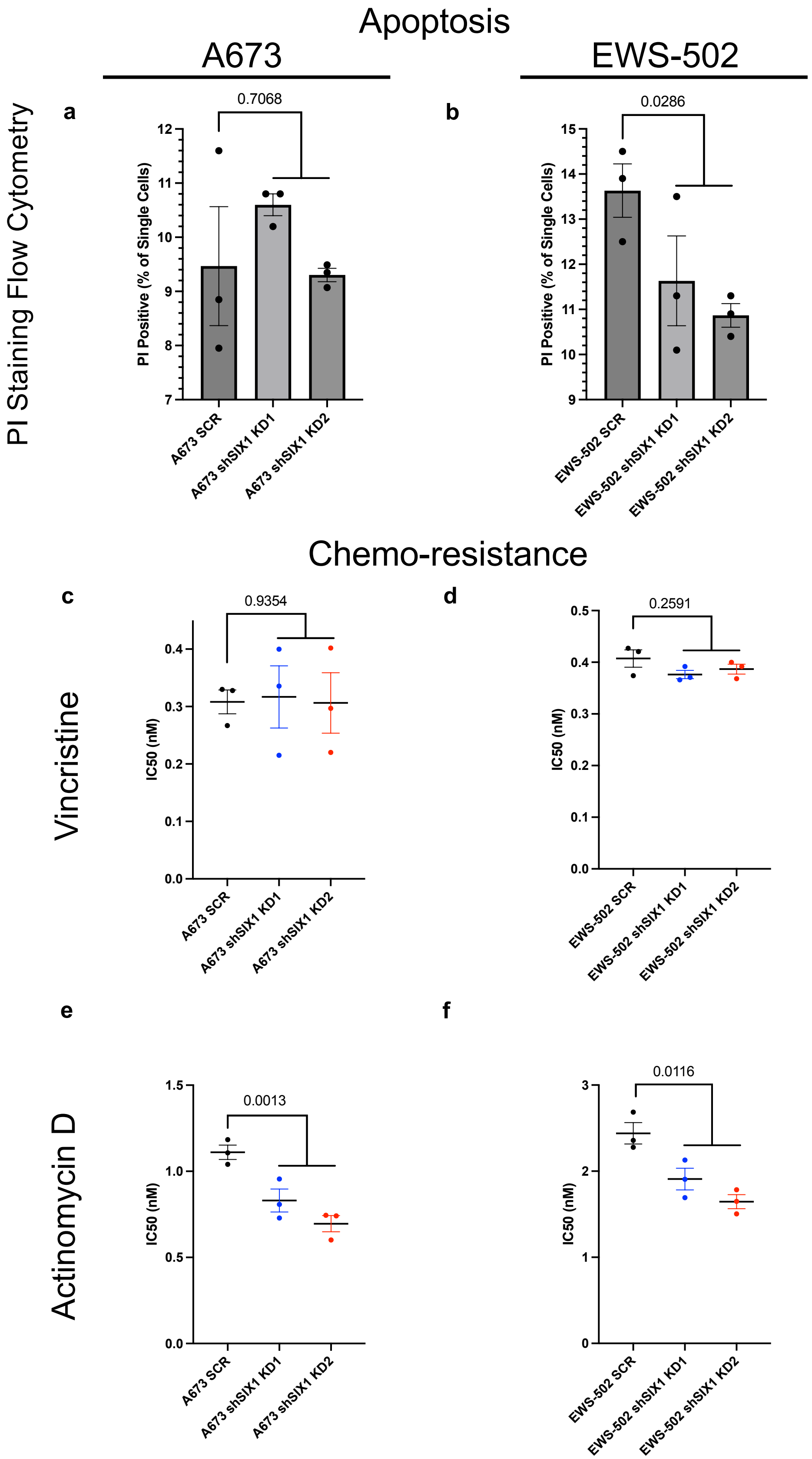

Supplementary Figure 4. SIX1 KD leads to mixed effects on apoptosis and chemo-resistance in A673 and EWS-502 cells. Propidium iodide staining flow cytometry for SCR and shSIX1 KD cells in the (a) A673 and (b) EWS-502 systems. Mean±SEM of n=3 independent experimental replicates shown. Statistical analysis performed between SCR and combined SIX1 KD replicates using unpaired two-tailed Welch's T-test. Chemo-resistance to Vincristine (as measured by IC50) for SCR and SIX1 KD cells in the (c) A673 and (d) EWS-502 systems. Chemo-resistance to Actinomycin D (as measured by IC50) for SCR and SIX1 KD cells in the (e) A673 and (f) EWS-502 systems. Mean±SEM of n=3 independent experimental replicates shown. Each chemoresistance experimental replicate consists of n=5 technical replicates (averaged). Statistical analysis performed between SCR and combined SIX1 KD replicates using unpaired two-tailed Welch's T-test. Source data are available as a Source Data file.

a

A673

Pathway	pG	pGFDR
Pertussis	0.00011	0.02153
Calcium signaling pathway	0.00023	0.02369
Insulin secretion	0.00183	0.09034
Dilated cardiomyopathy (DCM)	0.00186	0.09034
Glutamatergic synapse	0.0022	0.09034
Axon guidance	0.00288	0.09842
Arrhythmogenic right ventricular cardiomyopathy (ARVC)	0.00401	0.11293
ECM-receptor interaction	0.00443	0.11293
Phospholipase D signaling pathway	0.00496	0.11293
Neuroactive ligand-receptor interaction	0.00558	0.11448

b

EWS-502

Pathway	pG	pGFDR
Gastric acid secretion	0.00045	0.06336
Gap junction	0.00062	0.06336
Focal adhesion	0.00101	0.06527
Calcium signaling pathway	0.00132	0.06527
Oxytocin signaling pathway	0.00161	0.06527
Insulin secretion	0.00332	0.11244
Aldosterone-regulated sodium reabsorption	0.00394	0.11437
ECM-receptor interaction	0.00487	0.12369
Rap1 signaling pathway	0.00685	0.15118
Glutamatergic synapse	0.00745	0.15118

Supplementary Figure 5. KEGG Topology Pathway Analysis in A673 SIX1 KD vs Control Cells. (a) Top pathway hits from KEGG Topology analysis of A673 SIX1 KD vs. SCR RNA-sequencing data with corresponding pG and pGFDR values shown. (b) Top pathway hits from KEGG Topology analysis of EWS-502 SIX1 KD vs. SCR RNA-sequencing data with corresponding pG and pGFDR values shown.

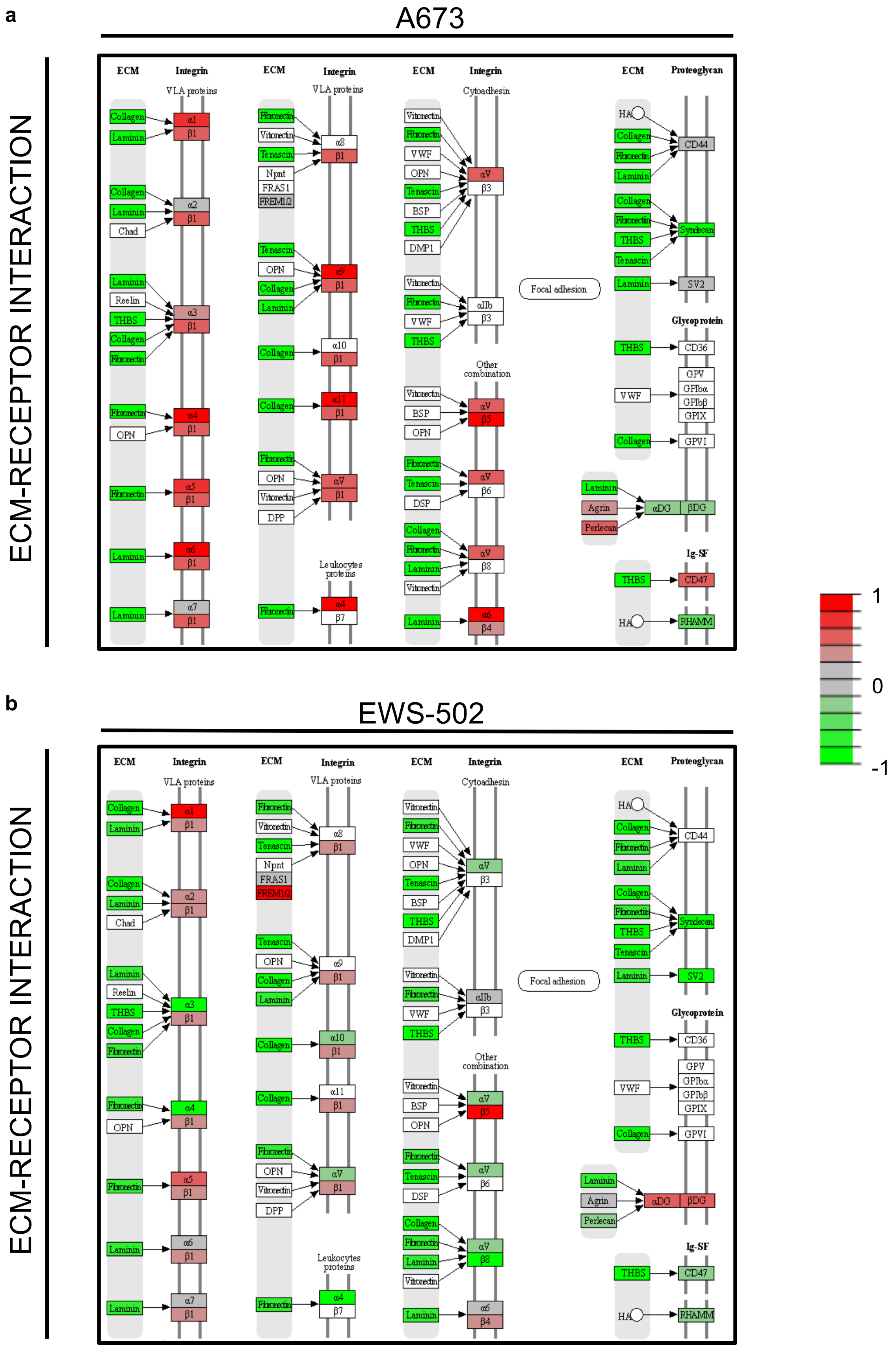

Supplementary Figure 6. SIX1 KD promotes expression of integrin genes in ECM-Receptor Interaction Kegg Pathway. ECM-Receptor Interaction Kegg Pathway comparing mRNA expression of shSIX1 KD1 to SCR cells for the (a) A673 and (b) EWS-502 cell lines.

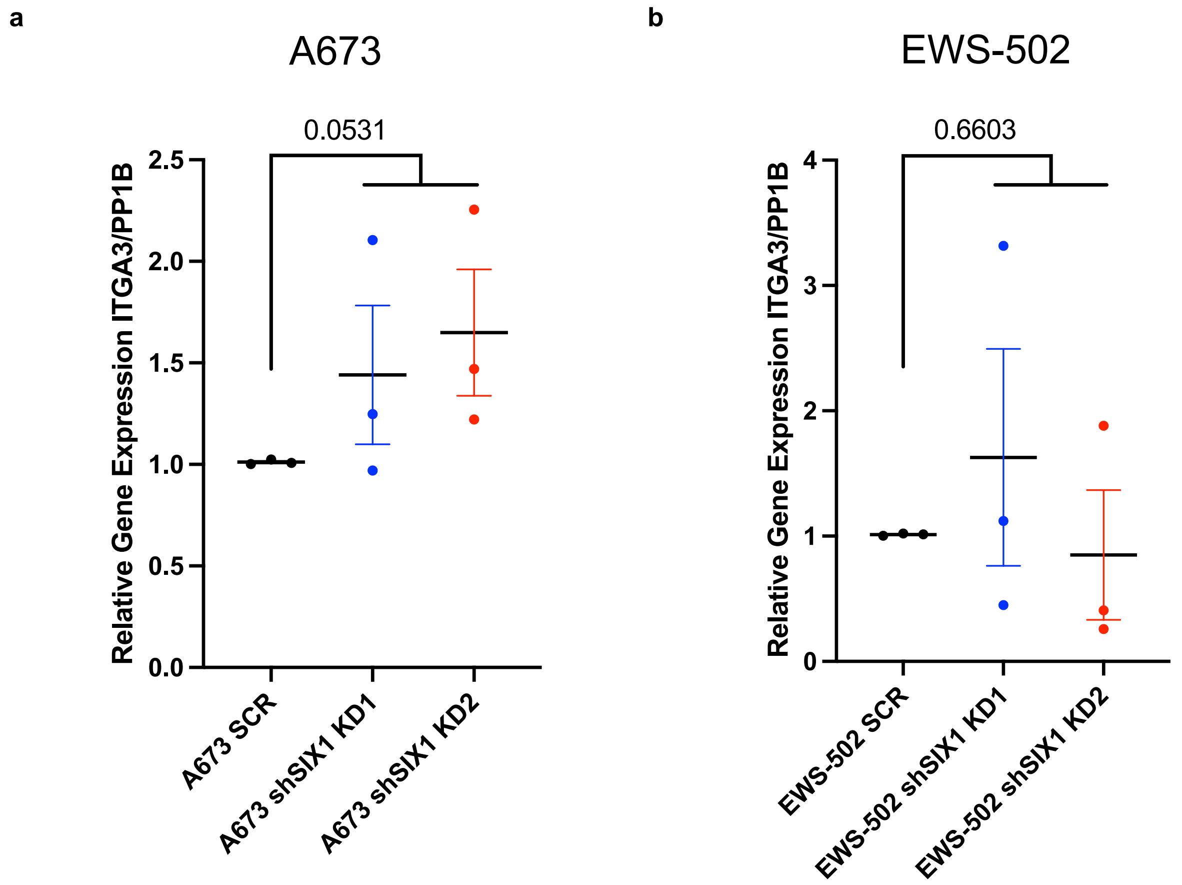

Supplementary Figure 7. SIX1 KD Does Not Consistently Increase ITGA3 Expression. (a) qRT-PCR analysis of ITGA3 gene expression in A673 SCR and SIX1 KD cells. (b) qRT-PCR analysis of ITGA3 gene expression in EWS-502 SCR and SIX1 KD cells. Mean±SEM of n=3 independent experimental replicates shown. Each experimental replicate consists of n=3 technical replicates (averaged). Statistical analysis performed between SCR and combined SIX1 KD replicates using unpaired two-tailed Welch's T-test. Source data are available as a Source Data file.

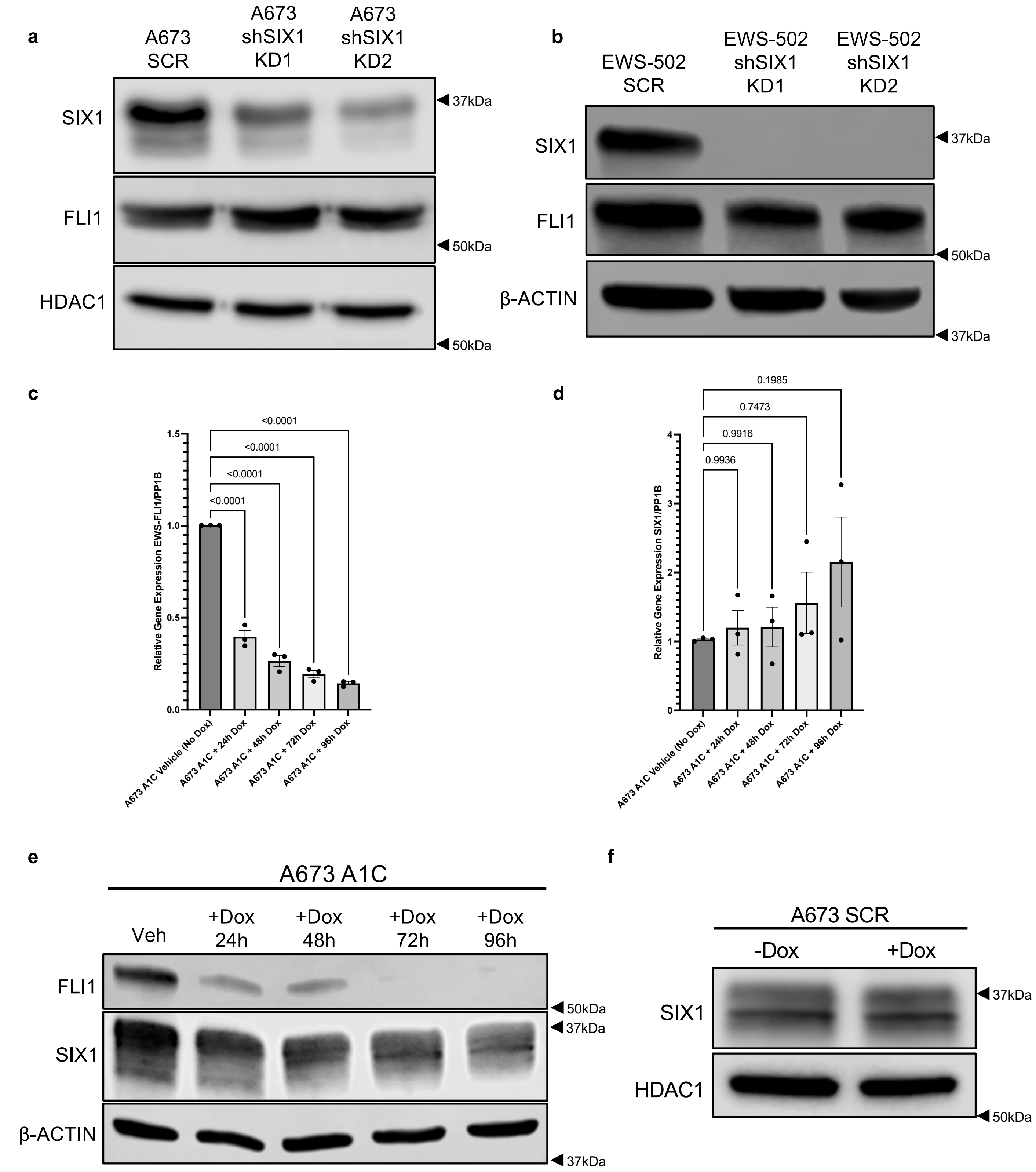

Supplementary Figure 8. SIX1 does not regulate EWS/FLI1 expression but EWS/FLI1 enhances SIX1 protein levels. Western blotting analysis for of SIX1 and FLI1 in (a) A673 SCR and SIX1 KD cells and (b) EWS-502 SCR and SIX1 KD cells. qRT-PCR analysis for (c) EWS/FLI1 expression and (d) SIX1 expression in A673 A1C (dox-inducible EWS/FLI1 KD) cells treated with the indicated time course of 2μg/ml doxycycline. Mean±SEM of n=3 independent experimental replicates shown. Each experimental replicate consists of n=3 technical replicates (averaged). Statistical analysis performed with a one-way ANOVA with post-hoc Dunnett's multiple comparisons test. (e) Western blotting analysis for FLI1 and SIX1 in time course of doxycycline treatment in A673 A1C cells. (f) Western blotting analysis for SIX1 levels in A673 SCR cell with 48h treatment with 2μg/ml doxycycline as a control for e. Representative Western blotting results shown for one of three independent experimental replicates. Source data are available as a Source Data file. Uncropped blots are available at the end of this Supplementary Data file.

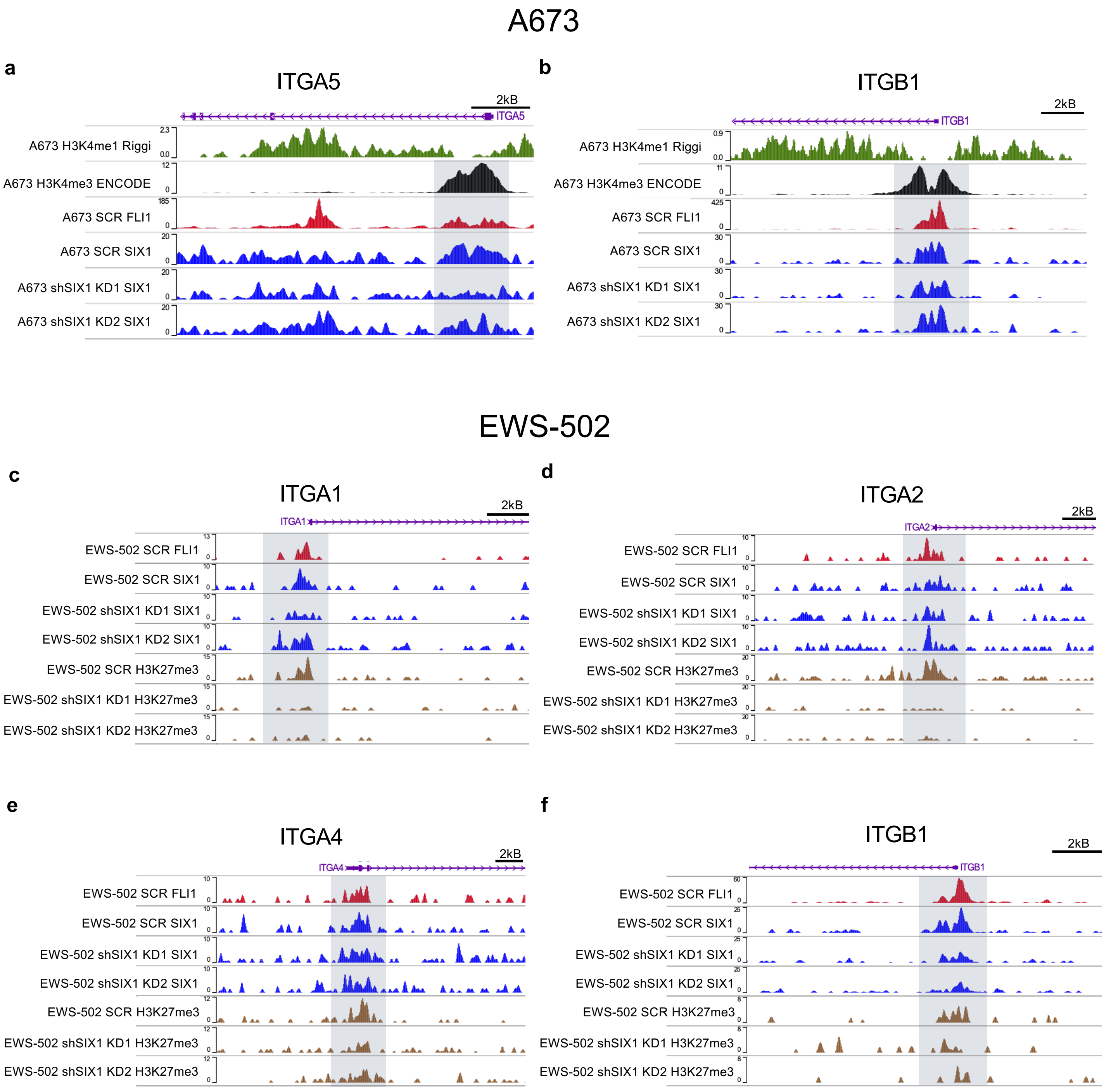

Supplementary Figure 9. SIX1 and EWS/FLI1 bind at integrin gene promoters in A673 and EWS-502 cells. CUT&RUN tracks for A673 SCR and shSIX1 KD cells around the promoter region of (a) ITGA5 and (b) ITGB1. Tracks shown are H3K4me1, H3K4me3, FLI1, and SIX1. CUT&RUN tracks for EWS-502 SCR and shSIX1 KD cells around the promoter region of (c) ITGA1, (d) ITGA2, (e) ITGA4, and (f) ITGB1. Tracks shown are FLI1, SIX1, and H3K27me3.

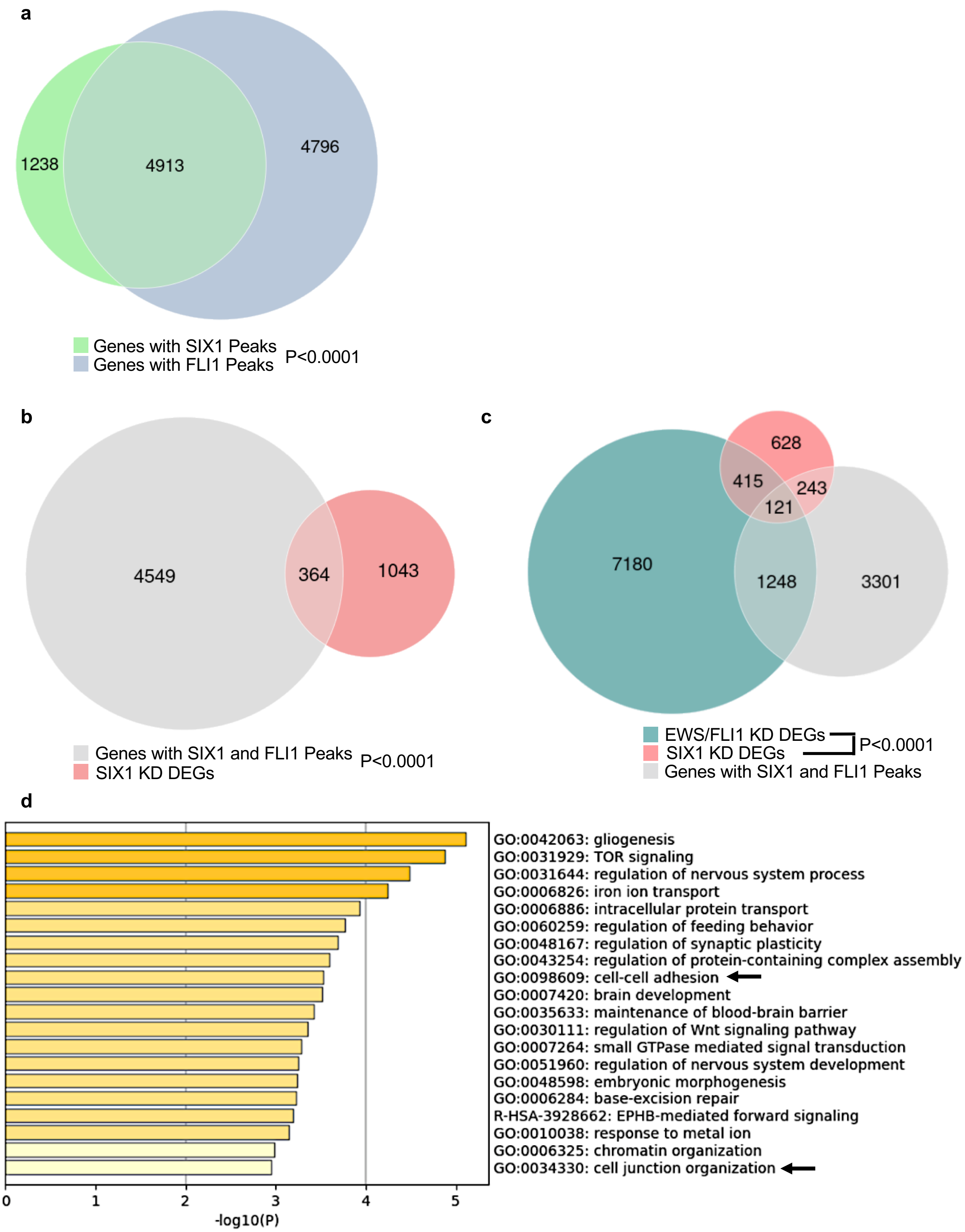

Supplementary Figure 10. SIX1 and EWS/FLI1 are co-bound and co-regulate overlapping gene sets in EWS-502 cells. (a) Venn diagram of genes bound by SIX1 and/or EWS/FLI1 in EWS-502 SCR cells. (b) Venn diagram of co-bound genes by SIX1 and EWS/FLI1 and DEGs with SIX1 KD in EWS-502 cells. (c) Venn diagram of co-bound genes, EWS-502 SIX1 KD DEGs, and A673 EWS/FLI1 KD DEGs. Statistics for all Venn diagrams performed using hypergeometric test (one-tailed Fisher's exact test). (d) Metascape enriched gene sets from the three-way overlapping genes from c. Statistics for pathways performed using hypergeometric test.

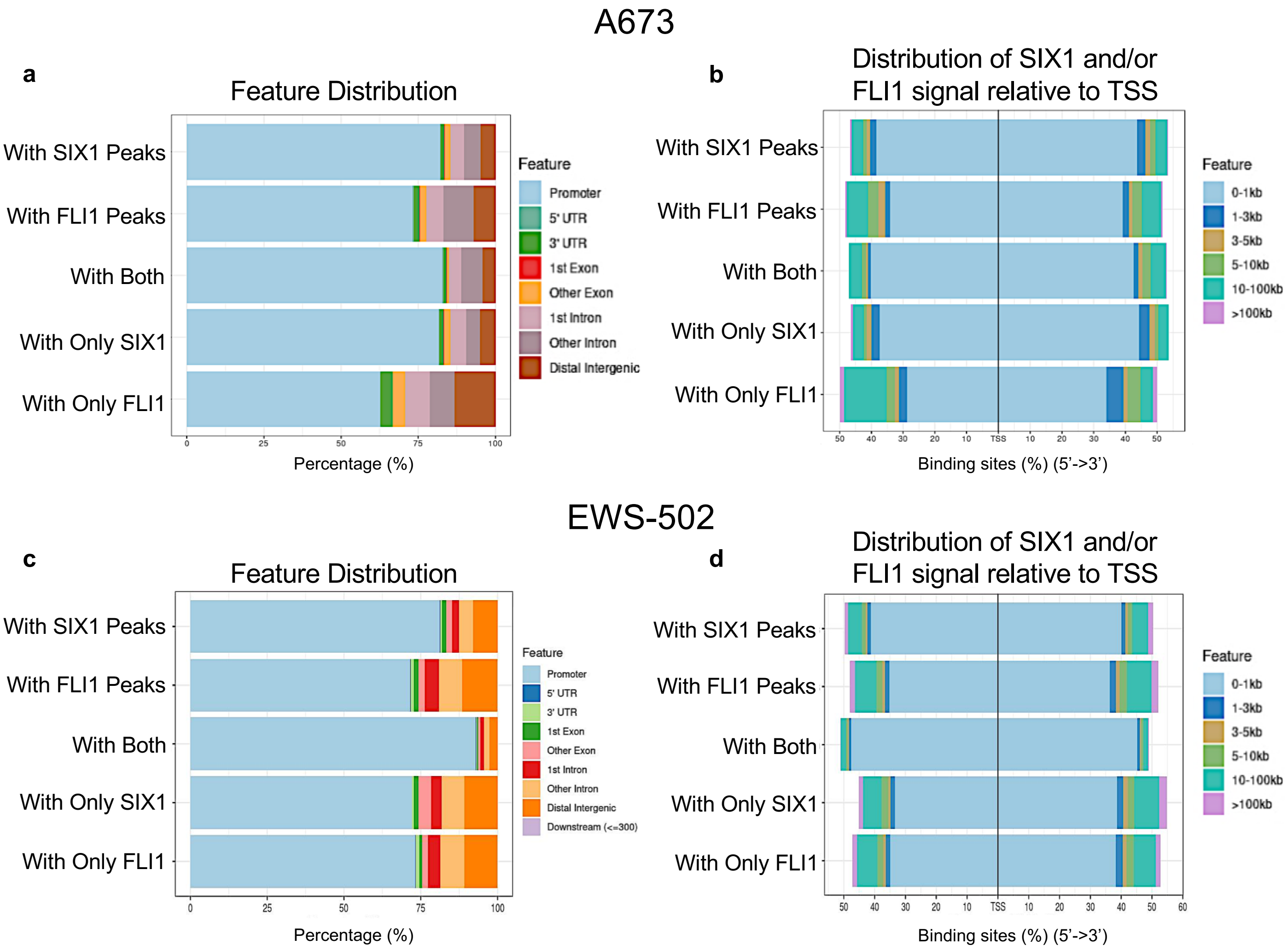

Supplementary Figure 11. SIX1 and EWS/FLI1 are predominantly co-bound at gene promoters. (a) Feature distribution and (b) Distance from TSS of SIX1 and FLI1 bound sites in A673 SCR CUT&RUN. (c) Feature distribution and (d) Distance from TSS of SIX1 and FLI1 bound sites in EWS-502 SCR CUT&RUN.

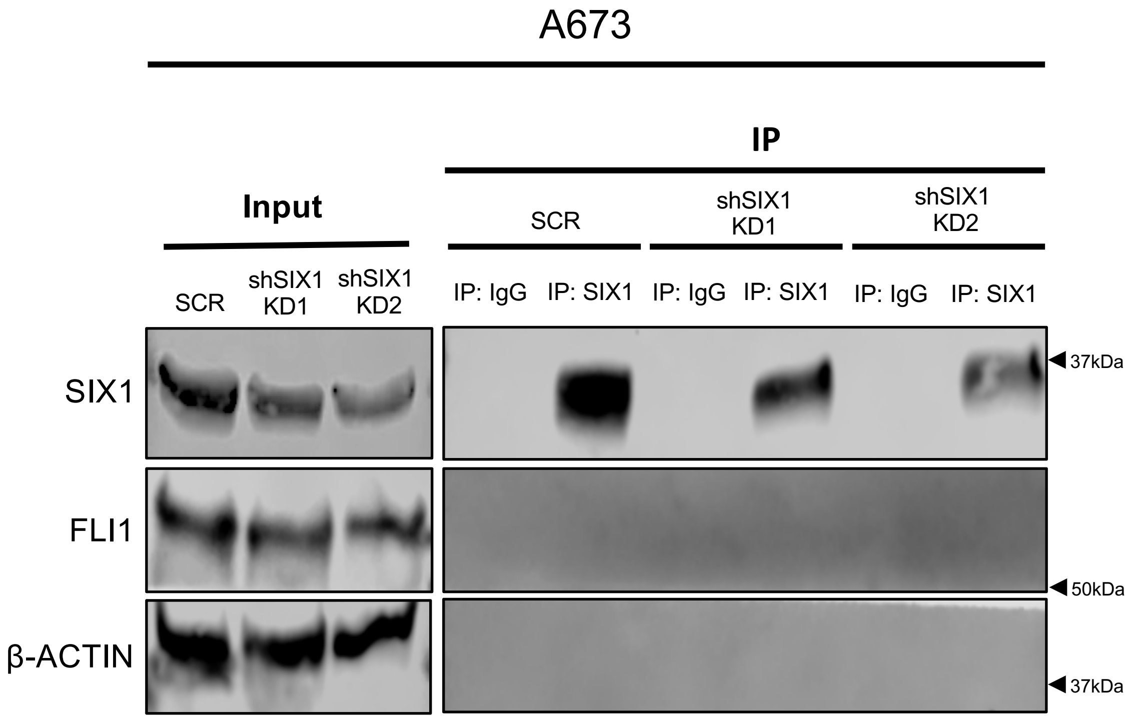

Supplementary Figure 12. SIX1 does not bind EWS/FLI1 in ES cells. Western blotting analysis of SIX1 and FLI1 after IgG or SIX1 immunoprecipitation in A673 SCR and SIX1 KD cells. Representative images shown for one of three independent experiments. Uncropped blots are available at the end of this Supplementary Data file.

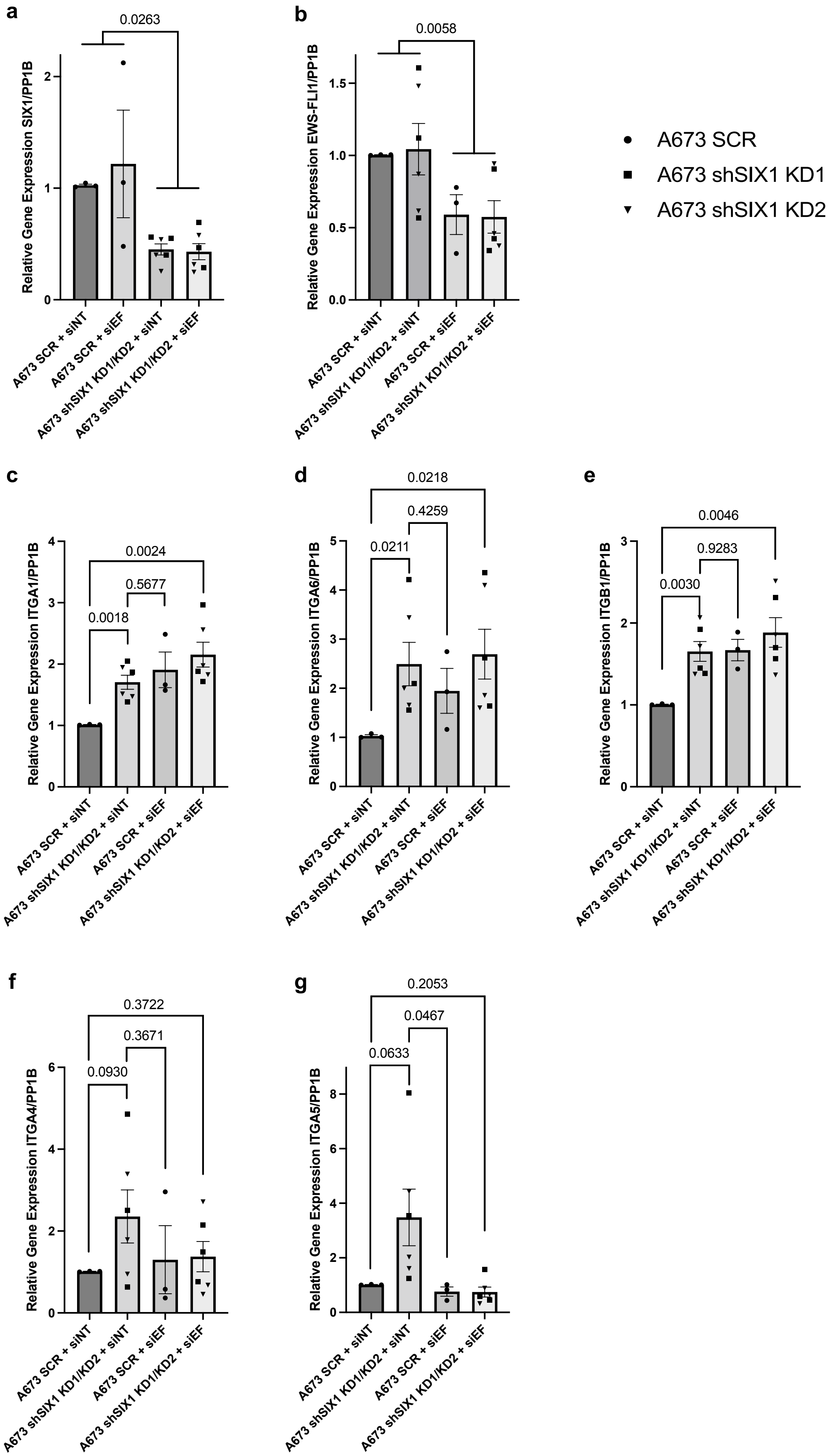

Supplementary Figure 13. SIX1 and EWS/FLI1 have complex roles in co-regulating integrin gene expression. Expression levels of (a) SIX1 and (b) EWS-FLI1 in A673 SCR or SIX1 KD cells with non-targeting siRNA (siNT) or siRNA-mediated EWS/FLI1 KD (siEF) via qRT-PCR. Statistical analysis performed between combined SCR/siNT groups and combined KD groups as indicated using unpaired two-tailed Welch's T-test. Gene expression levels of (c) ITGA1, (d) ITGA6, (e) ITGB1, (f) ITGA4, and (g) ITGA5 via qRT-PCR for the treatment groups described for a. and b. Mean±SEM of n=3 independent experimental replicates shown (SCR groups n=3/group, SIX1 KD groups n=6/group – n=3 of each KD). Each experimental replicate consists of n=3 technical replicates (averaged). Statistical analysis performed with unpaired two-tailed Welch's T-test between indicated groups. Source data are available as a Source Data file.

Supplementary Table 1. Enriched known motifs at FLI1-bound sites in A673 CUT&RUN.
P-values calculated using hypergeometric test, with q-values calculated after false discovery rate correction by the Benjamini-Hochberg method.

Rank	Motif	Name	P-value	log P-pvalue	q-value (Benjamini)	# Target Sequences with Motif	% of Targets Sequences with Motif
1	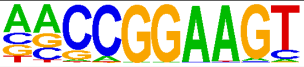	ETS(ETS)/Promoter/Homer	1e-137	-3.161e+02	0.0000	1472.0	24.05%
2	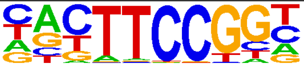	Elk1(ETS)/Hela-Elk1-ChIP-Seq(GSE31477)/Homer	1e-121	-2.795e+02	0.0000	2192.0	35.82%
3	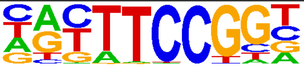	Elk4(ETS)/Hela-Elk4-ChIP-Seq(GSE31477)/Homer	1e-118	-2.738e+02	0.0000	2200.0	35.95%
4	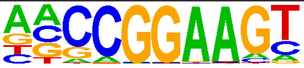	ELF1(ETS)/Jurkat-ELF1-ChIP-Seq(SRA014231)/Homer	1e-105	-2.419e+02	0.0000	1999.0	32.66%
5	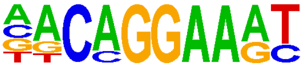	EWS:FLI1-fusion(ETS)/SK_N_MC-EWS:FLI1-ChIP-Seq(SRA014231)/Homer	1e-100	-2.310e+02	0.0000	1365.0	22.30%
6	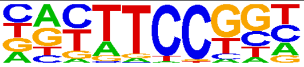	Fli1(ETS)/CD8-FLI-ChIP-Seq(GSE20898)/Homer	1e-98	-2.264e+02	0.0000	2635.0	43.06%
7	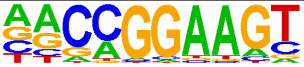	GABPA(ETS)/Jurkat-GABPa-ChIP-Seq(GSE17954)/Homer	1e-97	-2.234e+02	0.0000	2150.0	35.13%
8	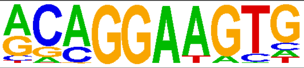	ETS1(ETS)/Jurkat-ETS1-ChIP-Seq(GSE17954)/Homer	1e-79	-1.842e+02	0.0000	2119.0	34.62%
9	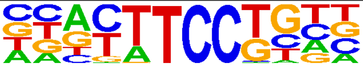	Etv2(ETS)/ES-ER71-ChIP-Seq(GSE59402)/Homer	1e-75	-1.731e+02	0.0000	1752.0	28.63%
10	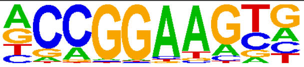	ETV4(ETS)/HepG2-ETV4-ChIP-Seq(ENCODE)/Homer	1e-74	-1.718e+02	0.0000	2770.0	45.26%
11	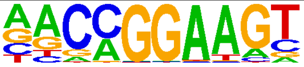	ETV1(ETS)/GIST48-ETV1-ChIP-Seq(GSE22441)/Homer	1e-70	-1.628e+02	0.0000	2608.0	42.61%
12	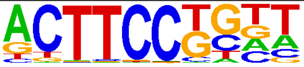	Elf4(ETS)/BMDM-Elf4-ChIP-Seq(GSE88699)/Homer	1e-66	-1.537e+02	0.0000	1953.0	31.91%
13	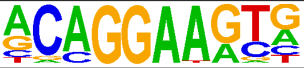	ERG(ETS)/VCaP-ERG-ChIP-Seq(GSE14097)/Homer	1e-50	-1.158e+02	0.0000	2458.0	40.16%
14	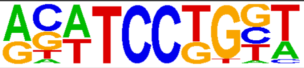	SPDEF(ETS)/VCaP-SPDEF-ChIP-Seq(SRA014231)/Homer	1e-33	-7.817e+01	0.0000	1508.0	24.64%
15	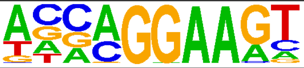	EHF(ETS)/LoVo-EHF-ChIP-Seq(GSE49402)/Homer	1e-33	-7.671e+01	0.0000	1789.0	29.23%
16	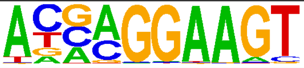	ELF5(ETS)/T47D-ELF5-ChIP-Seq(GSE30407)/Homer	1e-33	-7.667e+01	0.0000	1135.0	18.55%
17	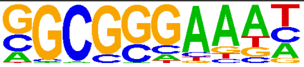	E2F4(E2F)/K562-E2F4-ChIP-Seq(GSE31477)/Homer	1e-22	-5.258e+01	0.0000	2028.0	33.14%
18	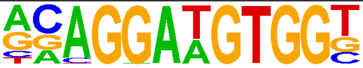	ETS:RUNX(ETS,Runt)/Jurkat-RUNX1-ChIP-Seq(GSE17954)/Homer	1e-22	-5.219e+01	0.0000	281.0	4.59%
19	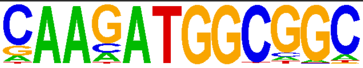	YY1(Zf)/Promoter/Homer	1e-21	-4.956e+01	0.0000	347.0	5.67%
20	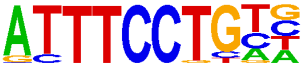	EWS:ERG-fusion(ETS)/CADO_ES1-EWS:ERG-ChIP-Seq(SRA014231)/Homer	1e-18	-4.308e+01	0.0000	664.0	10.85%

Supplementary Table 2. Enriched known motifs at SIX1-bound sites in A673 CUT&RUN.
P-values calculated using hypergeometric test, with q-values calculated after false discovery rate correction by the Benjamini-Hochberg method.

Rank	Motif	Name	P-value	log P-pvalue	q-value (Benjamini)	# Target Sequences with Motif	% of Targets Sequences with Motif
1	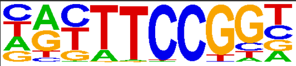	Elk4(ETS)/Hela-Elk4-ChIP-Seq(GSE31477)/Homer	1e-48	-1.120e+02	0.0000	3755.0	33.83%
2	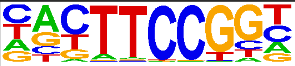	Elk1(ETS)/Hela-Elk1-ChIP-Seq(GSE31477)/Homer	1e-41	-9.629e+01	0.0000	3702.0	33.35%
3	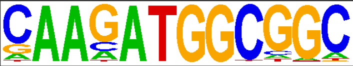	YY1(Zf)/Promoter/Homer	1e-41	-9.496e+01	0.0000	762.0	6.86%
4	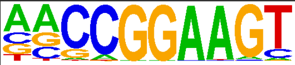	ETS(ETS)/Promoter/Homer	1e-36	-8.475e+01	0.0000	2145.0	19.32%
5	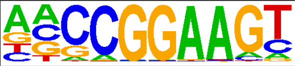	ELF1(ETS)/Jurkat-ELF1-ChIP-Seq(SRA014231)/Homer	1e-35	-8.067e+01	0.0000	3354.0	30.22%
6	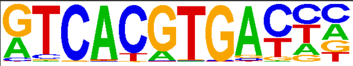	TFE3(bHLH)/MEF-TFE3-ChIP-Seq(GSE75757)/Homer	1e-28	-6.643e+01	0.0000	466.0	4.20%
7	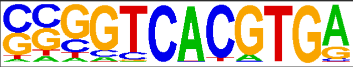	E-box(bHLH)/Promoter/Homer	1e-27	-6.222e+01	0.0000	667.0	6.01%
8	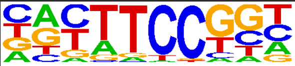	Fli1(ETS)/CD8-FLI-ChIP-Seq(GSE20898)/Homer	1e-26	-6.202e+01	0.0000	4573.0	41.20%
9	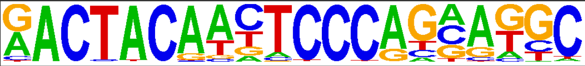	Ronin(THAP)/ES-Thap11-ChIP-Seq(GSE51522)/Homer	1e-21	-4.942e+01	0.0000	445.0	4.01%
10	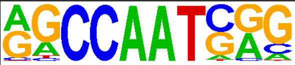	NFY(CCAAT)/Promoter/Homer	1e-20	-4.736e+01	0.0000	2235.0	20.14%
11	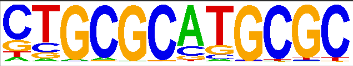	NRF1(NRF)/MCF7-NRF1-ChIP-Seq(Unpublished)/Homer	1e-20	-4.707e+01	0.0000	2029.0	18.28%
12	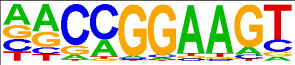	GABPA(ETS)/Jurkat-GABPa-ChIP-Seq(GSE17954)/Homer	1e-19	-4.487e+01	0.0000	3529.0	31.79%
13	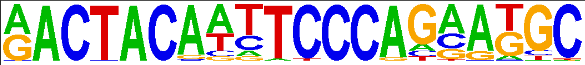	GFY-Staf(?,Zf)/Promoter/Homer	1e-19	-4.450e+01	0.0000	493.0	4.44%
14	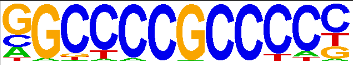	Sp1(Zf)/Promoter/Homer	1e-17	-4.036e+01	0.0000	4209.0	37.92%
15	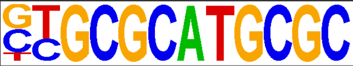	NRF(NRF)/Promoter/Homer	1e-17	-4.019e+01	0.0000	1822.0	16.41%
16	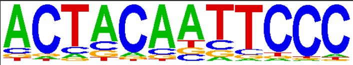	GFY(?)/Promoter/Homer	1e-17	-4.016e+01	0.0000	480.0	4.32%
17	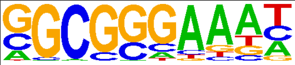	E2F4(E2F)/K562-E2F4-ChIP-Seq(GSE31477)/Homer	1e-14	-3.282e+01	0.0000	4136.0	37.26%
18	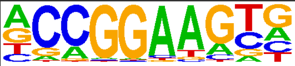	ETV4(ETS)/HepG2-ETV4-ChIP-Seq(ENCODE)/Homer	1e-13	-3.120e+01	0.0000	4849.0	43.68%
19	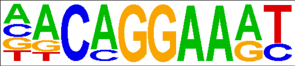	EWS:FLI1-fusion(ETS)/SK_N_MC-EWS:FLI1-ChIP-Seq(SRA014231)/Homer	1e-13	-3.039e+01	0.0000	1946.0	17.53%
20	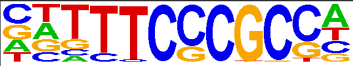	E2F7(E2F)/Hela-E2F7-ChIP-Seq(GSE32673)/Homer	1e-12	-2.845e+01	0.0000	1333.0	12.01%

Supplementary Table 3. Enriched known motifs at FLI1-bound sites in EWS-502 CUT&RUN.
P-values calculated using hypergeometric test, with q-values calculated after false discovery rate correction by the Benjamini-Hochberg method.

Rank	Motif	Name	P-value	log P-pvalue	q-value (Benjamini)	# Target Sequences with Motif	% of Targets Sequences with Motif
1	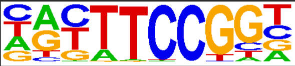	Elk4(ETS)/Hela-Elk4-ChIP-Seq(GSE31477)/Homer	1e-188	-4.343e+02	0.0000	2318.0	23.87%
2	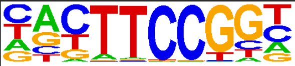	Elk1(ETS)/Hela-Elk1-ChIP-Seq(GSE31477)/Homer	1e-188	-4.340e+02	0.0000	2318.0	23.87%
3	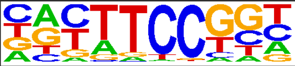	Fli1(ETS)/CD8-FLI-ChIP-Seq(GSE20898)/Homer	1e-164	-3.789e+02	0.0000	2738.0	28.20%
4	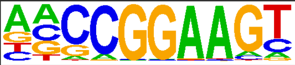	ELF1(ETS)/Jurkat-ELF1-ChIP-Seq(SRA014231)/Homer	1e-153	-3.538e+02	0.0000	2057.0	21.19%
5	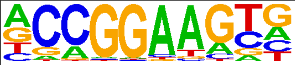	ETV4(ETS)/HepG2-ETV4-ChIP-Seq(ENCODE)/Homer	1e-151	-3.481e+02	0.0000	2954.0	30.43%
6	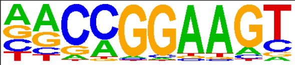	GABPA(ETS)/Jurkat-GABPa-ChIP-Seq(GSE17954)/Homer	1e-148	-3.411e+02	0.0000	2145.0	22.09%
7	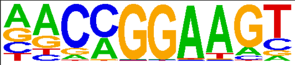	ETV1(ETS)/GIST48-ETV1-ChIP-Seq(GSE22441)/Homer	1e-147	-3.391e+02	0.0000	2695.0	27.76%
8	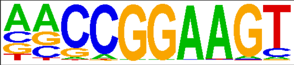	ETS(ETS)/Promoter/Homer	1e-144	-3.317e+02	0.0000	1414.0	14.56%
9	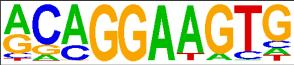	ETS1(ETS)/Jurkat-ETS1-ChIP-Seq(GSE17954)/Homer	1e-112	-2.595e+02	0.0000	2050.0	21.11%
10	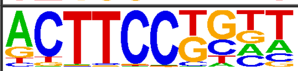	Elf4(ETS)/BMDM-Elf4-ChIP-Seq(GSE88699)/Homer	1e-110	-2.542e+02	0.0000	1929.0	19.87%
11	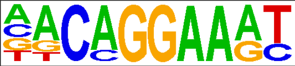	EWS:FLI1-fusion(ETS)/SK_N_MC-EWS:FLI1-ChIP-Seq(SRA014231)/Homer	1e-102	-2.369e+02	0.0000	1236.0	12.73%
12	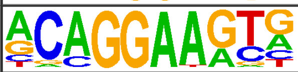	ERG(ETS)/VCaP-ERG-ChIP-Seq(GSE14097)/Homer	1e-99	-2.280e+02	0.0000	2404.0	24.76%
13	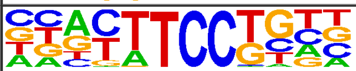	Etv2(ETS)/ES-ER71-ChIP-Seq(GSE59402)/Homer	1e-97	-2.256e+02	0.0000	1643.0	16.92%
14	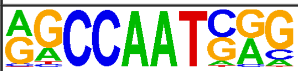	NFY(CCAAT)/Promoter/Homer	1e-62	-1.440e+02	0.0000	1104.0	11.37%
15	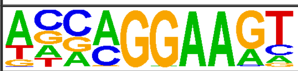	EHF(ETS)/LoVo-EHF-ChIP-Seq(GSE49402)/Homer	1e-60	-1.396e+02	0.0000	1673.0	17.23%
16	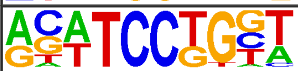	SPDEF(ETS)/VCaP-SPDEF-ChIP-Seq(SRA014231)/Homer	1e-55	-1.270e+02	0.0000	1343.0	13.83%
17	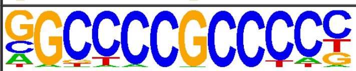	Sp1(Zf)/Promoter/Homer	1e-45	-1.048e+02	0.0000	2124.0	21.88%
18	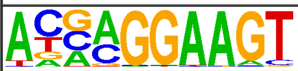	ELF5(ETS)/T47D-ELF5-ChIP-Seq(GSE30407)/Homer	1e-44	-1.026e+02	0.0000	1010.0	10.40%
19	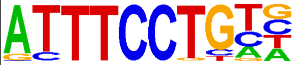	EWS:ERG-fusion(ETS)/CADO_ES1-EWS:ERG-ChIP-Seq(SRA014231)/Homer	1e-28	-6.628e+01	0.0000	576.0	5.93%
20	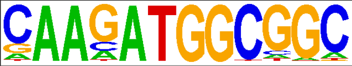	YY1(Zf)/Promoter/Homer	1e-27	-6.392e+01	0.0000	343.0	3.53%

Supplementary Table 4. Enriched known motifs at SIX1-bound sites in EWS-502 CUT&RUN.
P-values calculated using hypergeometric test, with q-values calculated after false discovery rate correction by the Benjamini-Hochberg method.

Rank	Motif	Name	P-value	log P-pvalue	q-value (Benjamini)	# Target Sequences with Motif	% of Targets Sequences with Motif
1	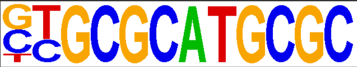	NRF(NRF)/Promoter/Homer	1e-114	-2.628e+02	0.0000	1442.0	23.44%
2	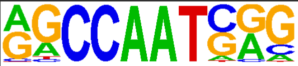	NFY(CCAAT)/Promoter/Homer	1e-97	-2.244e+02	0.0000	1499.0	24.37%
3	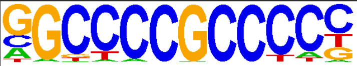	Sp1(Zf)/Promoter/Homer	1e-96	-2.226e+02	0.0000	2660.0	43.25%
4	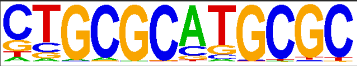	NRF1(NRF)/MCF7-NRF1-ChIP-Seq(Unpublished)/Homer	1e-93	-2.152e+02	0.0000	1507.0	24.50%
5	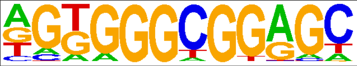	Sp5(Zf)/mES-Sp5.Flag-ChIP-Seq(GSE72989)/Homer	1e-70	-1.615e+02	0.0000	4063.0	66.05%
6	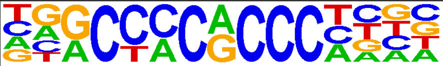	KLF3(Zf)/MEF-Klf3-ChIP-Seq(GSE44748)/Homer	1e-68	-1.572e+02	0.0000	2505.0	40.73%
7	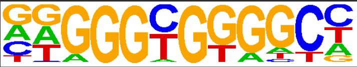	KLF1(Zf)/HUDEP2-KLF1-CutnRun(GSE136251)/Homer	1e-65	-1.511e+02	0.0000	3623.0	58.90%
8	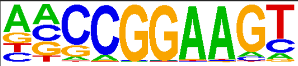	ELF1(ETS)/Jurkat-ELF1-ChIP-Seq(SRA014231)/Homer	1e-56	-1.299e+02	0.0000	1778.0	28.91%
9	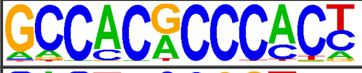	Klf9(Zf)/GBM-Klf9-ChIP-Seq(GSE62211)/Homer	1e-56	-1.299e+02	0.0000	1971.0	32.04%
10	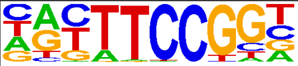	Elk4(ETS)/Hela-Elk4-ChIP-Seq(GSE31477)/Homer	1e-55	-1.272e+02	0.0000	1935.0	31.46%
11	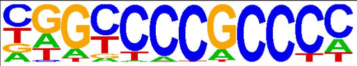	Sp2(Zf)/HEK293-Sp2.eGFP-ChIP-Seq(Encode)/Homer	1e-53	-1.237e+02	0.0000	4631.0	75.29%
12	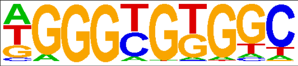	KLF5(Zf)/LoVo-KLF5-ChIP-Seq(GSE49402)/Homer	1e-50	-1.164e+02	0.0000	4157.0	67.58%
13	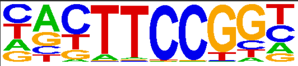	Elk1(ETS)/Hela-Elk1-ChIP-Seq(GSE31477)/Homer	1e-50	-1.154e+02	0.0000	1895.0	30.81%
14	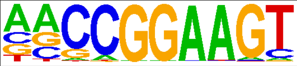	ETS(ETS)/Promoter/Homer	1e-46	-1.063e+02	0.0000	1137.0	18.48%
15	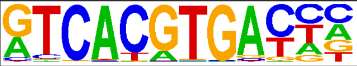	TFE3(bHLH)/MEF-TFE3-ChIP-Seq(GSE75757)/Homer	1e-46	-1.062e+02	0.0000	324.0	5.27%
16	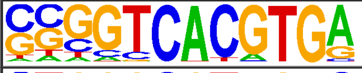	E-box(bHLH)/Promoter/Homer	1e-41	-9.608e+01	0.0000	434.0	7.06%
17	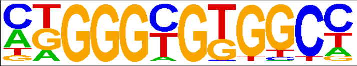	KLF6(Zf)/PDAC-KLF6-ChIP-Seq(GSE64557)/Homer	1e-40	-9.312e+01	0.0000	3653.0	59.39%
18	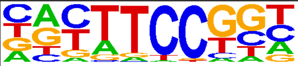	Fli1(ETS)/CD8-FLI-ChIP-Seq(GSE20898)/Homer	1e-38	-8.782e+01	0.0000	2278.0	37.03%
19	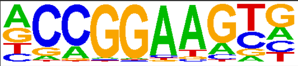	ETV4(ETS)/HepG2-ETV4-ChIP-Seq(ENCODE)/Homer	1e-37	-8.729e+01	0.0000	2496.0	40.58%
20	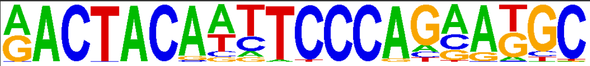	GFY-Staf(?,Zf)/Promoter/Homer	1e-35	-8.195e+01	0.0000	315.0	5.12%

Supplementary Table 5. Primer sequences. All primer names and sequences used for qRT-PCR experiments.

Primer Name	Primer Sequence
**Human SIX1 FWD**	TGCGCCGAAAATTTCCA
**Human SIX1 REV**	TTGAAGCAGTAGCTGGTCTCC
**Human EWS/FLI11 FWD**	TCCTACAGCCAAGCTCCAAGTCAA
**Human EWS/FLI11 REV**	AGGAGGAATTGCCACAGCTGGAT
**Human PPIB FWD**	GGAGATGGCACAGGAGGAAA
**Human PPIB REV**	CGTAGTGCTTCAGTTTGAAGT
**Human ITGA1 FWD**	TTCTTCTAGTCGGAGCCCCT
**Human ITGA1 REV**	TGTGCATGAATTGTGCTGCC
**Human ITGA2 FWD**	AGTGGCTTTCCTGAGAACCG
**Human ITGA2 REV**	CTGGTGAGGATCAAGCCGAG
**Human ITGA3 FWD**	CTGCACCCCAAAAACATCACC
**Human ITGA3 REV**	CTGCCACCCATCATTGTTCA
**Human ITGA4 FWD**	TGGAGTGCCCCCTGATTTAC
**Human ITGA4 REV**	ACAAAAAGAGAGCCAGTCCAGT
**Human ITGA5 FWD**	GTCGGGGGCTTCAACTTAGAC
**Human ITGA5 REV**	ACAGAGGTAGACAGCACCAC
**Human ITGA6 FWD**	AGAGGGATGTGGAGACGACA
**Human ITGA6 REV**	TTGGGTTGGAAGGGCTGTTT
**Human ITGB1 FWD**	CCAACCTGATCCTGTGTCCC
**Human ITGB1 REV**	ACCATGACCTCGTTGTTCCC
**Human CDH1 FWD**	ATTTTTCCCTCGACACCCGAT
**Human CDH1 REV**	TCCCAGGCGTAGACCAAGA
**Human CDH2 FWD**	AGCCAACCTTAACTGAGGAGT
**Human CDH2 REV**	GGCAAGTTGATTGGAGGGATG
**Human TWIST1 FWD**	GCCGGAGACCTAGATGTCATT
**Human TWIST1 REV**	TTTTAAAAGTGCGCCCCACG
**Human SNAI1 FWD**	CTCTAGGCCCTGGCTGCTA
**Human SNAI1 REV**	TCTTGACATCTGAGTGGGTCTG
**Human FN1 FWD**	GAGTGTGTGTGTCTTGGTAATGG
**Human FN1 REV**	CCACGTTTCTCCGACCAC
**Human VIM FWD**	AGTCCACTGAGTACCGGAGAC
**Human VIM REV**	CATTTCACGCATCTGGCGTTC

Supplementary Table 6. Primary antibody reagent information. All primary antibodies with relevant information used in Western blotting assays.

Reagent – Primary Antibodies	Source	Identifier	Clone Number (if applicable)	Dilution Used
**Mouse monoclonal anti-SIX1 992**	In-house purified antibody	N/A	N/A	1:500
**Rabbit polyclonal anti-SIX1**	Sigma	HPA001893	N/A	1:500
**Rabbit polyclonal anti-SIX1**	Cell Signaling Technology	D4A8K	N/A	1:1000
**Rabbit polyclonal anti-FLI1**	Abcam	ab15289	N/A	1:500
**Rabbit monoclonal anti-FLI1**	Abcam	ab133485	EPR4646	1:500
**Mouse monoclonal anti-β-Actin**	Sigma	A5316	AC-74	1:5000
**Mouse monoclonal anti-HDAC1**	Santa Cruz Biotechnology	Sc-81598	10E2	1:200
**Rabbit polyclonal anti-Integrin β1**	Cell Signaling Technology	4706S	N/A	1:1000
**Rabbit monoclonal anti-integrin α1**	Cell Signaling Technology	15574S	E9K2J	1:1000
**Rabbit monoclonal anti-integrin α2**	Invitrogen	MA5-32306	SN0752	1:1000
**Mouse monoclonal anti-integrin α4**	Santa Cruz Biotechnology	Sc-365209	A-7	1:1000
**Goat polyclonal anti-integrin α5**	R&D Systems	AF1864	N/A	1:1000
**Rabbit polyclonal anti-integrin α6**	Cell Signaling Technology	3750S	N/A	1:1000
**Mouse monoclonal anti-FAK**	Invitrogen	AHO1272	34Q36	1:500
**Rabbit monoclonal anti-p-FAK (Y397)**	Abcam	ab81298	EP2160Y	1:1000
**Mouse monoclonal anti-SRC**	Invitrogen	AHO1152	184Q20	1:500
**Rabbit monoclonal anti-p-SRC (Y416)**	Cell Signaling Technology	6943S	D49G4	1:1000
**Rabbit polyclonal anti-NaKATPase**	Cell Signaling Technology	3010S	N/A	1:1000
**Normal Rabbit IgG**	Cell Signaling Technology	2729	N/A	1:1000

Supplementary Table 7. Secondary antibody reagent information. All secondary antibodies with relevant information used in Western blotting assays.

Reagent – Secondary Antibody	Source	Identifier	Dilution Used
**HRP-conjugated goat anti-mouse**	Li-Cor	926-80010	1:10000
**HRP-conjugated goat anti-rabbit**	Li-Cor	926-80011	1:10000
**HRP-conjugated donkey anti-goat**	Santa Cruz Biotechnology	Sc-2020	1:10000
**HRP-conjugated rabbit TrueBlot anti-rabbit**	Rockland Immunochemicals	18-8816-31	1:1000

Supplementary Table 8. Neutralizing antibody reagent information. All neutralizing antibodies with relevant information used in integrin neutralization invasion assays.

Neutralizing Antibody	Source	Identifier	Clone Number (if applicable)	Concentration Used
**Rat monoclonal anti-integrin β1**	BD Pharmingen	552828	Mab 13	0.6μg/ml
**Mouse monoclonal anti-integrin α1**	EMD Millipore	05-246	5E8D9	5μg/mL
**Mouse monoclonal anti-integrin α2/β1**	Abcam	Ab24697	P1E6	5μg/mL
**Mouse monoclonal anti-integrin α4**	R&D Systems	BBA37	2B4	5μg/mL
**Goal polyclonal anti-integrin α5**	R&D Systems	AF1864	N/A	20μg/mL

Supplementary Table 9. Primary antibody reagent information for CUT&RUN. All antibodies with relevant information used in CUT&RUN assays.

Antibody	Source	Identifier
**Rabbit polyclonal anti-IgG**	Epicypher	13-0042k
**Rabbit polyclonal anti-SIX1**	Sigma	HPA001893
**Rabbit monoclonal anti-FLI1**	Abcam	ab133485, clone EPR4646
**Rabbit polyclonal anti-H3K27me3**	Diagenode	C15410069

Uncropped Supplementary Figure Western blots

Supp Fig1a

Anti-HDAC1 75 kDa 50 kDa

Anti-SIX1 37 kDa

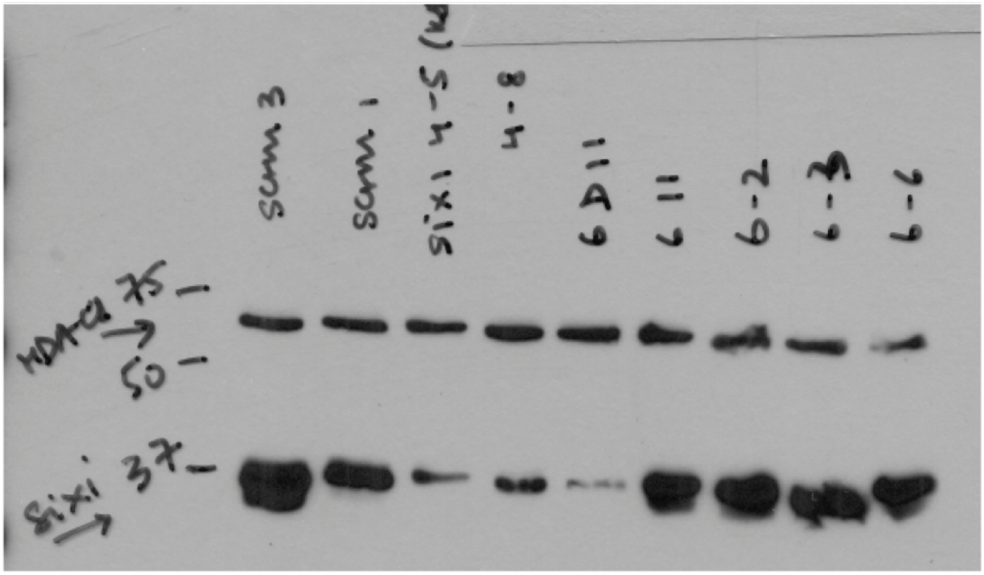

Supp Fig1b

Anti-SIX1

Anti-B-Actin

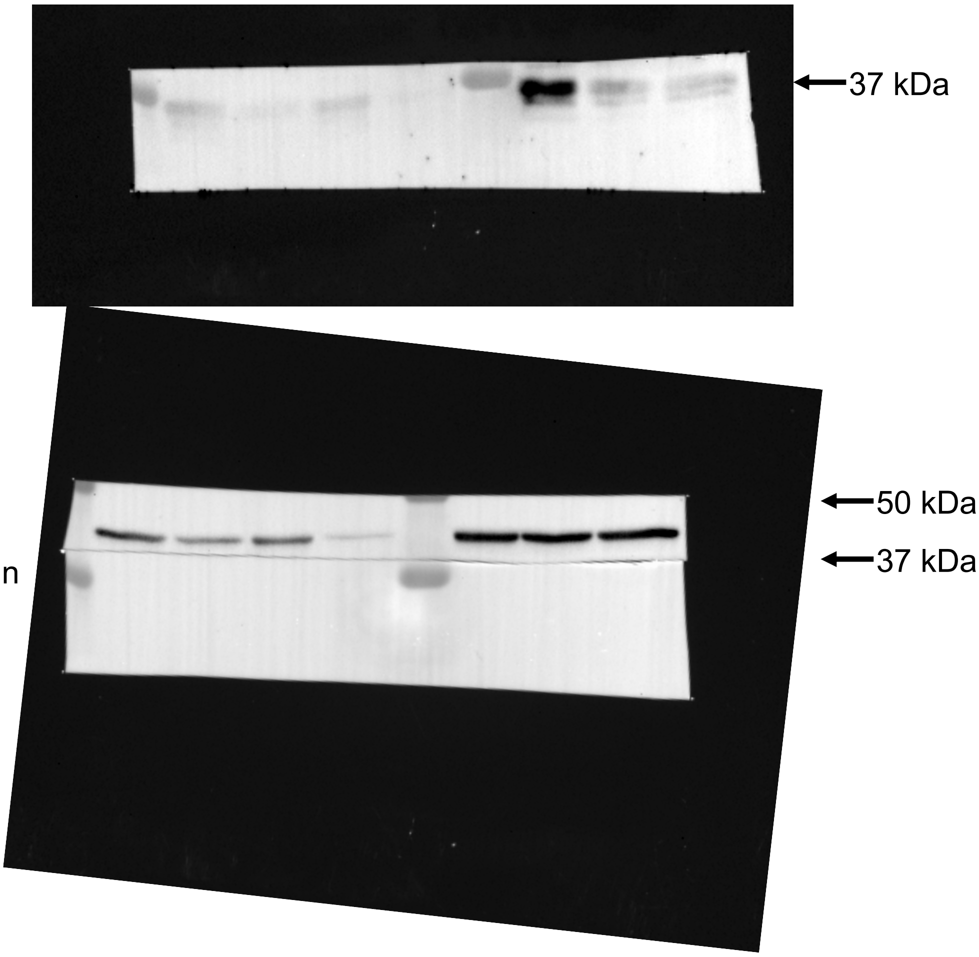

37 kDa

50 kDa

37 kDa

Uncropped Supplementary Figure Western blots

Supp Fig8a

Anti-SIX1

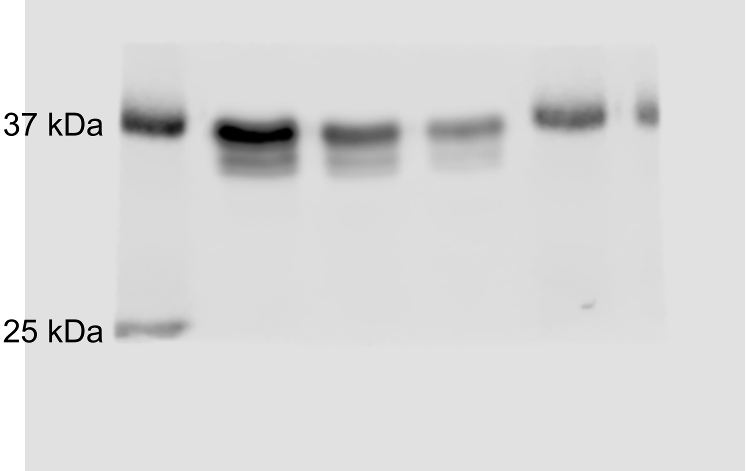

Anti-FLI1

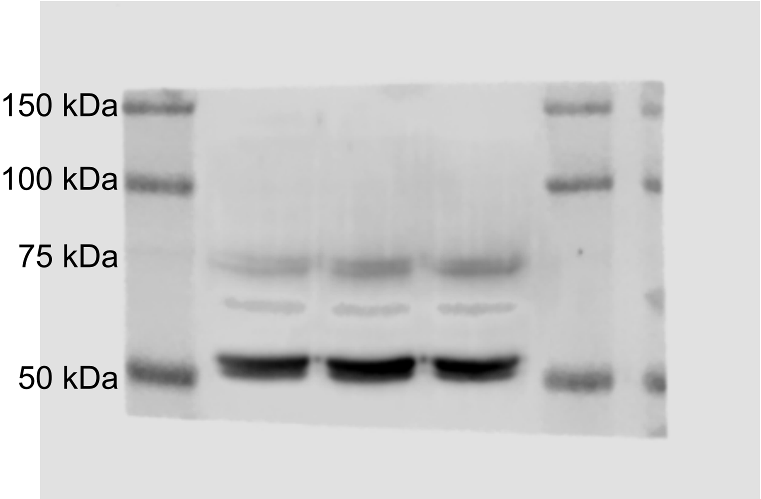

Anti-HDAC1

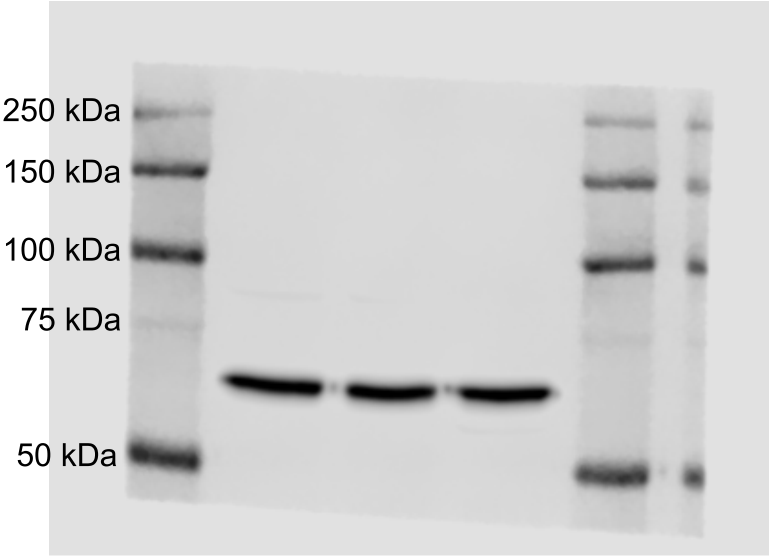

Uncropped Supplementary Figure Western blots

Supp Fig8b

Anti-SIX1

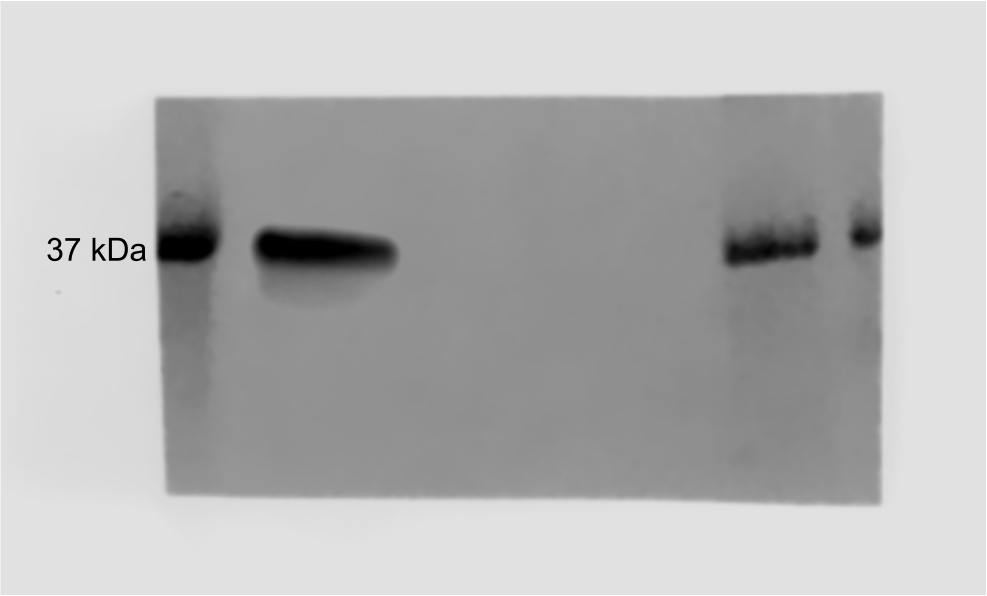

Anti-FLI1

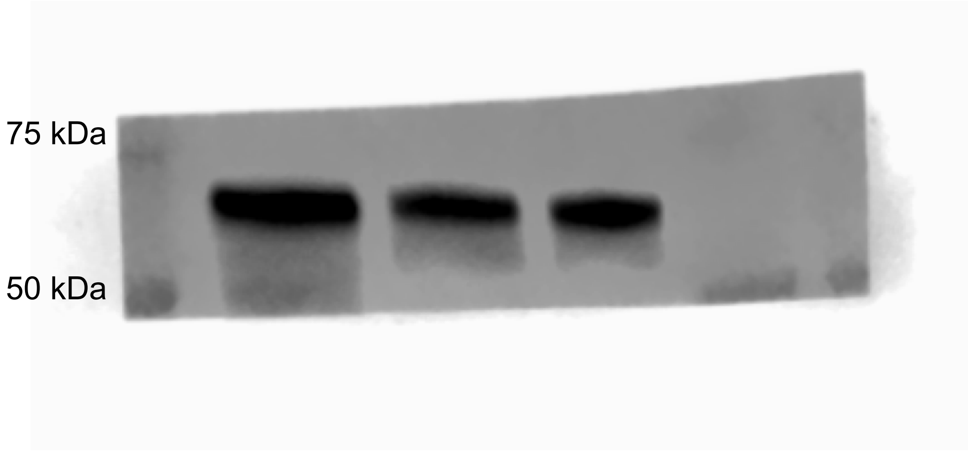

Anti-β-Actin

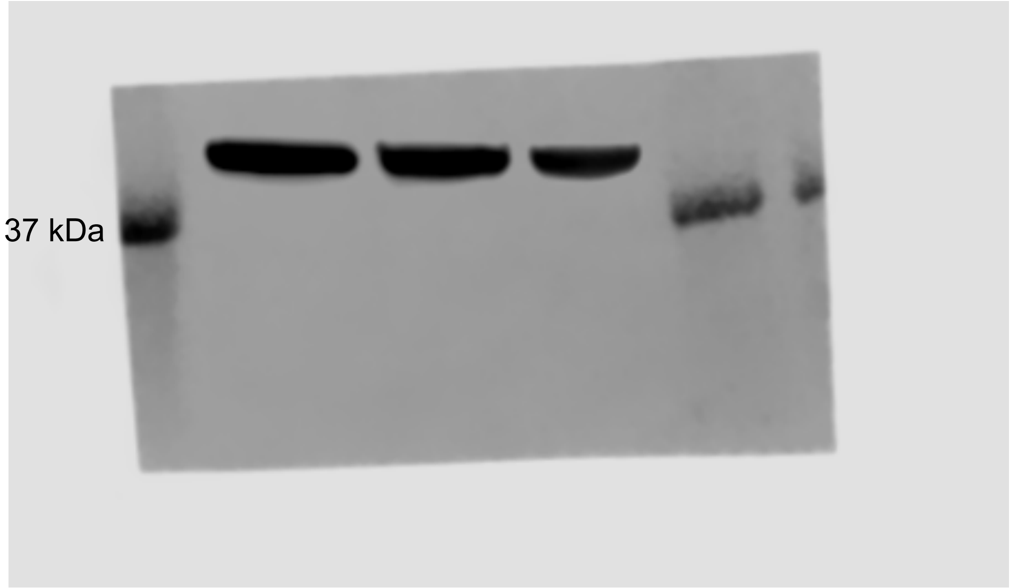

Uncropped Supplementary Figure Western blots

Supp Fig8e

Anti-FLI1

150 kDa
100 kDa
75 kDa
50 kDa

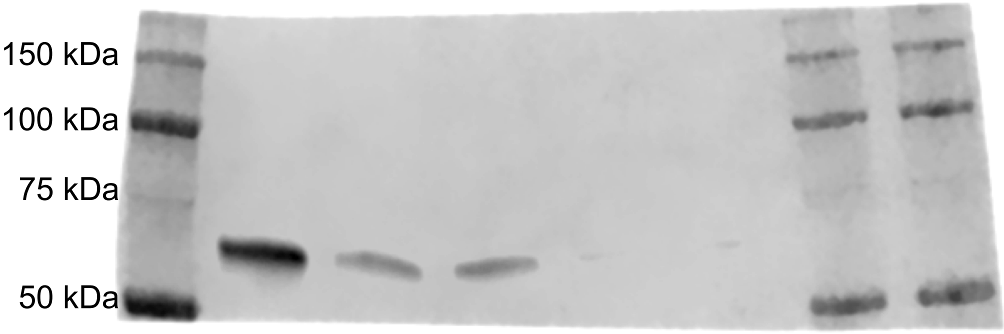

37 kDa
Anti-SIX1

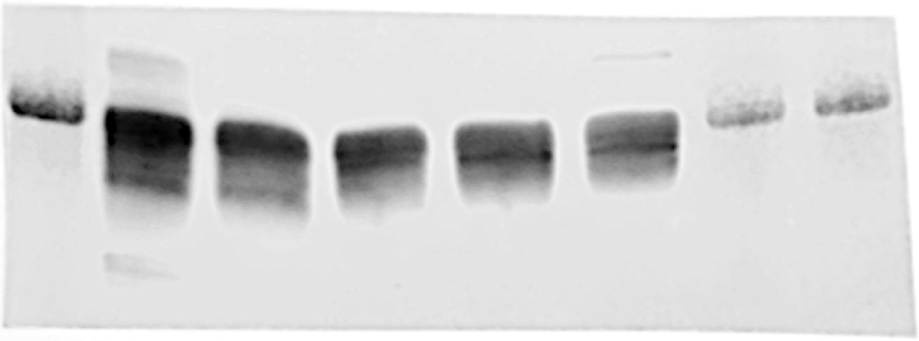

Anti-β-ACTIN

37 kDa

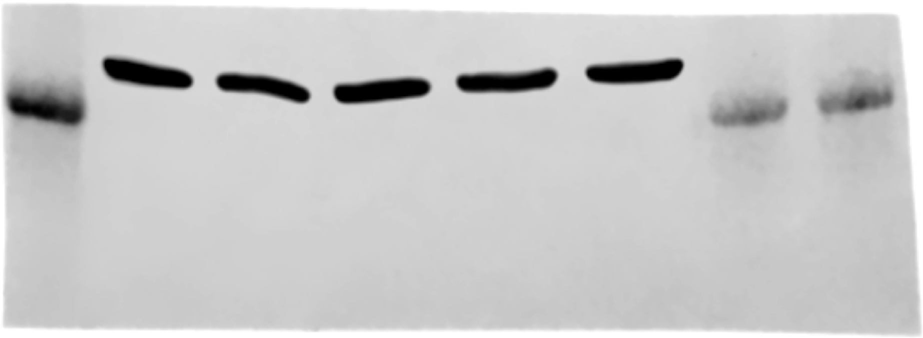

Uncropped Supplementary Figure Western blots

Supp Fig8f

Anti-SIX1

50 kDa
37 kDa

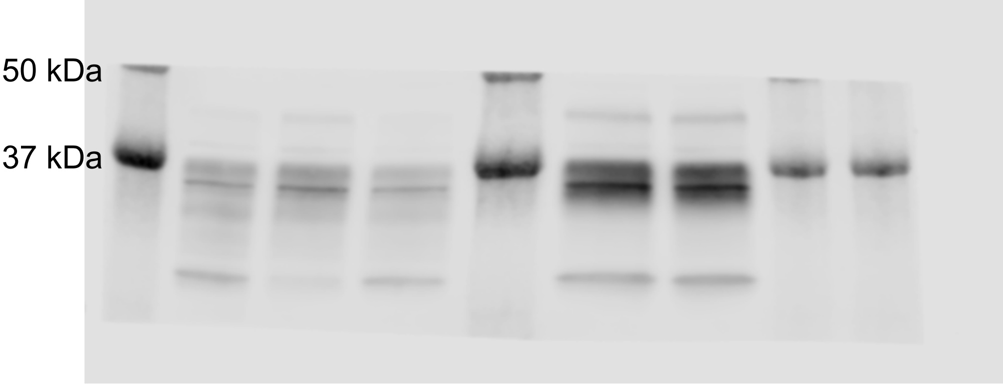

Anti-HDAC1

250 kDa
150 kDa
100 kDa
75 kDa
50 kDa

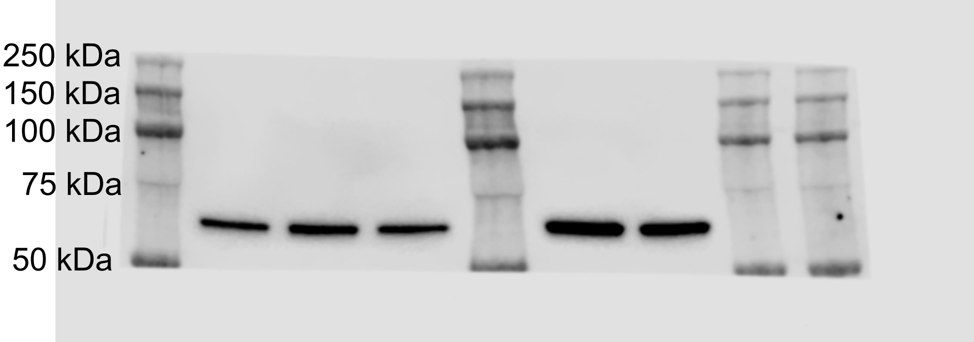

Uncropped Supplementary Figure Western blots

Supp Fig12a

Anti-SIX1

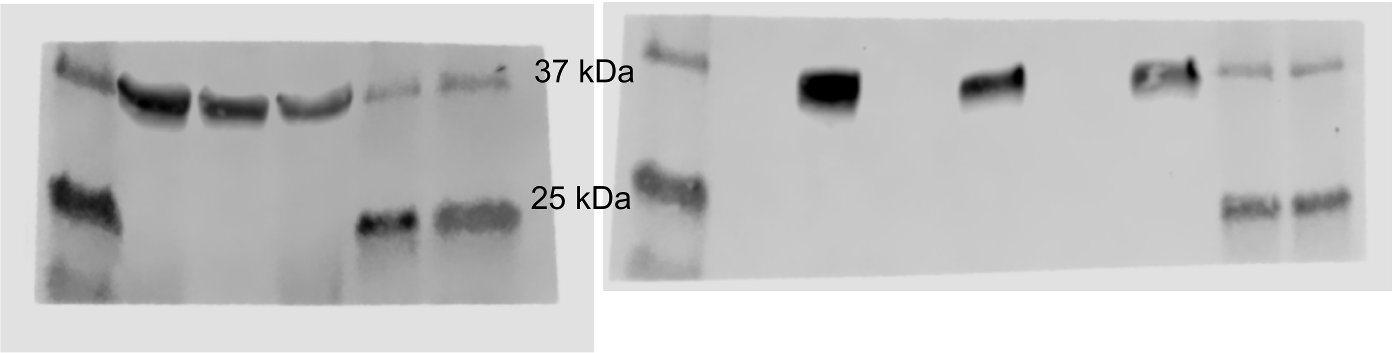

Anti-FLI1

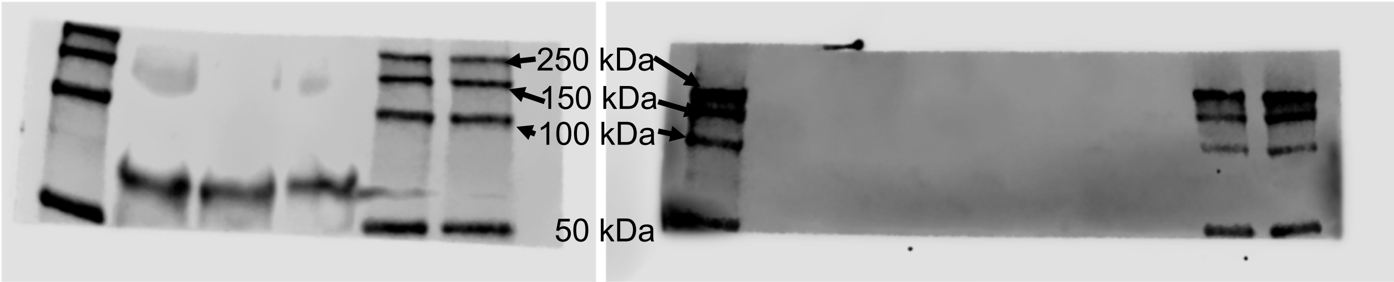

Anti-β-ACTIN

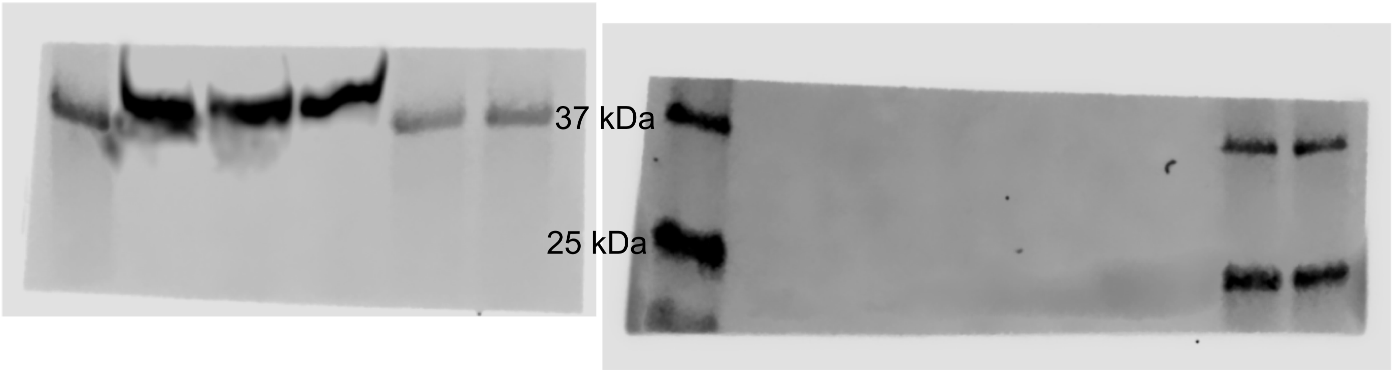

Supplement: Supplementary file 1 — Supplementary Information [file 41467_2023_39945_MOESM1_ESM.pdf]
