# Supplementary material for: SIX1 and EWS/FLI1 co-regulate an anti-metastatic gene network in Ewing Sarcoma
Source: Nat Commun. 2023 Jul 19;14:4357. doi: 10.1038/s41467-023-39945-w (PMC10356808; doi:10.1038/s41467-023-39945-w)
Supplement: Supplementary file 3 — Reporting Summary [file 41467_2023_39945_MOESM3_ESM.pdf]

nature portfolio

Corresponding author(s): Heide L Ford

Last updated by author(s): Jun 25, 2023

Reporting Summary

Nature Portfolio wishes to improve the reproducibility of the work that we publish. This form provides structure for consistency and transparency in reporting. For further information on Nature Portfolio policies, see our Editorial Policies and the Editorial Policy Checklist.

Statistics

For all statistical analyses, confirm that the following items are present in the figure legend, table legend, main text, or Methods section.

n/a	Confirmed	
☐	☒	The exact sample size (*n*) for each experimental group/condition, given as a discrete number and unit of measurement
☐	☒	A statement on whether measurements were taken from distinct samples or whether the same sample was measured repeatedly
☐	☒	The statistical test(s) used AND whether they are one- or two-sided<br>*Only common tests should be described solely by name; describe more complex techniques in the Methods section.*
☐	☒	A description of all covariates tested
☐	☒	A description of any assumptions or corrections, such as tests of normality and adjustment for multiple comparisons
☐	☒	A full description of the statistical parameters including central tendency (e.g. means) or other basic estimates (e.g. regression coefficient) AND variation (e.g. standard deviation) or associated estimates of uncertainty (e.g. confidence intervals)
☐	☒	For null hypothesis testing, the test statistic (e.g. *F*, *t*, *r*) with confidence intervals, effect sizes, degrees of freedom and *P* value noted<br>*Give P values as exact values whenever suitable.*
☒	☐	For Bayesian analysis, information on the choice of priors and Markov chain Monte Carlo settings
☒	☐	For hierarchical and complex designs, identification of the appropriate level for tests and full reporting of outcomes
☒	☐	Estimates of effect sizes (e.g. Cohen's *d*, Pearson's *r*), indicating how they were calculated

Our web collection on statistics for biologists contains articles on many of the points above.

Software and code

Policy information about availability of computer code

Data collection	In vivo imaging data was collected using LivingImage (v. 4.5.2). Blot images were acquired using Image Studio (v. 5.2.5)
Data analysis	RStudio (v. 1.2.5033) was used for human data analysis and generation of survival curves using standard methods. Prism (v.9.0) was used for all statistical analyses with the exception of the linear mixed-model analyses (performed in R). In vivo imaging data was analyzed using LivingImage (v. 4.5.2). RNA-sequencing data was processed/analyzed using the following packages: The quality of the fastq files was assessed using FastQC (<http://www.bioinformatics.babraham.ac.uk/projects/fastqc) and MultiQC (Ewels, P. et al. 2016). Illumina adapters and low-quality reads were filtered out using BBDuk (http://jgi.doe.gov/data-and-tools/bb-tools). Trimmed fastqc files were aligned to the hg38 human reference genome and aligned counts per gene were quantified using STAR (Dobin, A. et al. 2013). Transcript expression levels were estimated with Salmon using inferential replicates (Patro, R. et al. 2017). Differential gene and transcript analyses were performed using the DESeq2 (Love, M. et al. 2014) and Swish (Zhu, A. et al. 2019) packages. After processing, the University of Colorado Biostatistics and Bioinformatics Shared Resource RNA-seq analysis tool was used to perform pathway analysis (https://bioinformatics.cuanschutz.edu/UC_RNA-seq). CUT&RUN data was processed/analyzed using the following packages: The quality of the fastq files was accessed using FastQC (<http://www.bioinformatics.babraham.ac.uk/projects/fastqc) and MultiQC (Ewels, P. et al. 2016). Illumina adapters and low quality reads were filtered out using BBDuk (http://jgi.doe.gov/data-and-tools/bb-tools). Bowtie2 (v.2.3.4.3) (Langmead, B. & Salzberg, S. L. 2012) was used to align the sequencing reads to the hg38 reference human genome. Samtools (v.1.11) (Li, H. et al. 2009) was used to select the mapped reads (samtools view -b - q 30) and sort the bam files. PCR duplicates were removed using Picard MarkDuplicates tool (http://broadinstitute.github.io/picard/) The normalization ratio for each sample was calculated by dividing the number of uniquely mapped human reads of the sample with the lowest number of reads by the number of uniquely mapped human reads of each sample. These normalization ratios were used to randomly sub-sample reads to obtain the same number of reads for each sample using using samtools view -s. Bedtools genomecov was used to create bedgraph files from the bam files (Quinlan, A. R. & Hall, I. M. 2010). Bigwig files were created using deepTools bamCoverage (Ramirez, F. et al. 2016) and visualized using IGV (Robinson J. T. et al. 2011). Peaks were called using MACS2 (v2.1.2) (Zhang, Y

et al. 2008) using ENCODE recommendations. IDR was used to identify the reproducible peaks between the replicates (Li, Q. et al. 2011). Further processing of the peak data was performed in R, using the following tools: valR (Riemondy, K. A. et al. 2017), and DiffBind (Stark, R. and Brown, G. 2011), and ngs.plot (Shen, L. et al. 2014). All motif analyses were performed using the findMotifsGenome.pl program in the HOMER package (v.4.101.1) (Heinz, S. et al. 2010). All migration and invasion images were analyzed using ImageJ (v1.53).

For manuscripts utilizing custom algorithms or software that are central to the research but not yet described in published literature, software must be made available to editors and reviewers. We strongly encourage code deposition in a community repository (e.g. GitHub). See the Nature Portfolio guidelines for submitting code & software for further information.

Data

Policy information about availability of data

All manuscripts must include a data availability statement. This statement should provide the following information, where applicable:

- Accession codes, unique identifiers, or web links for publicly available datasets
- A description of any restrictions on data availability
- For clinical datasets or third party data, please ensure that the statement adheres to our policy

Raw NGS data (RNA-sequencing, CUT&RUN) has been deposited into the Gene Expression Omnibus and are publicly available under the accession number GSE215416 (https://www.ncbi.nlm.nih.gov/geo/query/acc.cgi?acc=GSE215416). The data supporting the findings of this study are available within the paper and its Supplementary Information. Raw data for all experiments, with the exception of the large bioinformatics datasets (available on GEO) are provided in the Source Data file. Publicly available exon array data for 85 Ewing sarcoma human tumor samples (used in Fig. 9a) is available from the NCBI Gene Expression Omnibus (GEO ID: GSE63157 - https://www.ncbi.nlm.nih.gov/geo/query/acc.cgi?acc=gse63157). Uncropped Western blot images are provided in the Source Data file and in the Supplementary Data file.

Human research participants

Policy information about studies involving human research participants and Sex and Gender in Research.

Reporting on sex and gender	N/A
Population characteristics	N/A
Recruitment	N/A
Ethics oversight	N/A

Note that full information on the approval of the study protocol must also be provided in the manuscript.

Field-specific reporting

Please select the one below that is the best fit for your research. If you are not sure, read the appropriate sections before making your selection.

☒ Life sciences ☐ Behavioural & social sciences ☐ Ecological, evolutionary & environmental sciences

For a reference copy of the document with all sections, see nature.com/documents/nr-reporting-summary-flat.pdf

Life sciences study design

All studies must disclose on these points even when the disclosure is negative.

Sample size	For in vivo study, sample size was determined based on previous experience in our laboratory with this category of experiments. Specific sample sizes for in vivo experiments were based on prior work with SIX1 KD systems in sarcomas, specifically Hsu et al. 2022. All in vitro assays were performed with at least three technical replicates per experiment and three independent experiments. NGS experiments utilized three technical replicates per group.
Data exclusions	One mouse was excluded from A673 in vivo subcutaneous flank experiment after being determined to be a significant outlier via Grubbs' test (this is also disclosed in the manuscript and figure legend). No data was excluded from any other analyses.
Replication	All findings presented in this manuscript were confirmed through three biological replicate experiments, with the exception of the in vivo studies. For the A673 in vivo study, n=4-6 biological replicates were used per group (exact sample sizes listed in manuscript and below). Attempts at replicating the A673 in vivo study were successful. The EWS-502 in vivo study was performed twice, but had poor overall outgrowth on the first attempt due to the use of luciferase-tagged cells which had reduced fitness. As such, the results for EWS-502 represent one experiment, but it was performed with sufficient power to draw conclusions as a second model.
Randomization	For the A673 in vivo tail vein study, animals were randomly assigned to one of three treatment groups: A673 SCR, A673 shSIX1 KD1, or shSIX1 KD2 (n=6 per group). For the A673 in vivo subcutaneous flank study, animals were randomly assigned to one of three treatment groups: A673 SCR (n=6), A673 shSIX1 KD1 (n=5), or shSIX1 KD2 (n=4). For the EWS-502 in vivo study, animals were randomly assigned to one of three

treatment groups: EWS-502 SCR (n=5), EWS-502 shSIX1 KD1 (n=5), and EWS-502 shSIX1 KD2 (n=4 - one mouse died during injection).

Blinding

For all transwell migration/invasion experiments, groups were blinded during imaging and data analysis. During in vivo tail vein experiments, groups were blinded during injection. Groups were unblinded during imaging to image mice with other mice in the same experimental group. All images were processed and analyzed uniformly across treatment groups, so blinding was not required during analysis of in vivo imaging data. For EWS-502 tail vein experiment, groups were blinded during necropsy and gross examination. Blinding was not used in soft agar assaysm as all groups were treated uniformly during image acquisition and analysis. No blinding was used during NGS experiments, as there was uniform processing and data analysis. Blinding was not used/necessary in Western blot, qRT-PCR, proliferation, apoptosis assays, and drug sensitivity assays due to the nature of the assays and uniform treatment/data processing/analysis applied to all groups.

Reporting for specific materials, systems and methods

We require information from authors about some types of materials, experimental systems and methods used in many studies. Here, indicate whether each material, system or method listed is relevant to your study. If you are not sure if a list item applies to your research, read the appropriate section before selecting a response.

Materials & experimental systems

n/a	Involved in the study
☐	☒ Antibodies
☐	☒ Eukaryotic cell lines
☒	☐ Palaeontology and archaeology
☐	☒ Animals and other organisms
☒	☐ Clinical data
☒	☐ Dual use research of concern

Methods

n/a	Involved in the study
☒	☐ ChIP-seq
☒	☐ Flow cytometry
☒	☐ MRI-based neuroimaging

Antibodies

Antibodies used

Mouse monoclonal anti-SIX1 992 In-house purified antibody (1:500)
Rabbit polyclonal anti-SIX1 Sigma HPA001893 Lot 00009690 (1:500)
Rabbit polyclonal anti-SIX1 Cell Signaling Technology D4A8K Lot 3 (1:1000)
Rabbit polyclonal anti-FLI1 Abcam ab15289 (1:500)
Rabbit monoclonal anti-FLI1 Abcam ab133485, clone EPR4646 (1:500)
Mouse monoclonal anti- β-Actin Sigma A5316 Lot 0000127607, clone AC-74 (1:5000)
Mouse monoclonal anti-HDAC1 Santa Cruz Biotechnology Sc-81598 Lot G2619, clone 10E2 (1:200)
Rabbit polyclonal anti-Integrin B1 Cell Signaling Technology 4706S Lot 6 (1:1000)
Rabbit monoclonal anti-integrin A1 Cell Signaling Technology 15574S Lot 1, clone E9K2J (1:1000)
Rabbit monoclonal anti-integrin A2 Invitrogen MA5-32306, clone SNO752 (1:1000)
Mouse monoclonal anti-integrin A4 Santa Cruz Biotechnology Sc-365209 Lot B0321, clone A-7 (1:1000)
Goat polyclonal anti-integrin A5 R&D Systems AF1864 (1:1000)
Rabbit polyclonal anti-integrin A6 Cell Signaling Technology 3750S Lot 3 (1:1000)
Mouse monoclonal anti-FAK Invitrogen AHO1272, clone 34Q36 (1:500)
Rabbit monoclonal anti-p-FAK (Y397) Abcam ab81298, clone EP2160Y (1:1000)
Mouse monoclonal anti-SRC Invitrogen AHO1152, clone 184Q20 (1:500)
Rabbit monoclonal anti-p-SRC (Y416) Cell Signaling Technology 6943S, clone D49G4 (1:1000)
Rabbit polyclonal anti-NaKATPase, Cell Signaling 3010S (1:1000)
Normal Rabbit IgG Cell Signaling Technology 2729 Lot 10 (1:1000)
HRP-conjugated goat anti-mouse Li-Cor 926-80010 (1:10000)
HRP-conjugated goat anti-rabbit Li-Cor 926-80011 (1:10000)
HRP-conjugated donkey anti-goat Santa Cruz Biotechnology Sc-2020 (1:10000)
HRP-conjugated rabbit TrueBlot anti-rabbit Rockland Immunochemicals 18-8816-31 (1:1000)
Rat monoclonal anti-integrin B1 BD Pharmingen 552828 (neutralizing), clone Mab 13 (0.6ug/ml)
Mouse monoclonal anti-integrin A1 EMD Millipore 05-246 (neutralizing), clone 5E8D9 (5ug/ml)
Mouse monoclonal anti-integrin A2/B1 Abcam ab24697 (neutralizing), clone P1E6 (5ug/ml)
Mouse monoclonal anti-integrin A4 R&D Systems BBA37 (neutralizing), clone 2B4 (5ug/ml)
Goat polyclonal anti-integrin A5 R&D Systems AF1864 (neutralizing) (20ug/ml)
Rabbit polyclonal anti-IgG Epicypher 13-0042k (CUT&RUN)
Rabbit polyclonal anti-SIX1 Sigma HPA001893 (CUT&RUN)
Rabbit monoclonal anti-FLI1 Abcam ab133485 (CUT&RUN), clone EPR4646
Rabbit polyclonal anti-H3K27me3 Diagenode C15410069 (CUT&RUN)

Validation

Mouse monoclonal anti-SIX1 992 In-house purified antibody, rabbit polyclonal anti-SIX1 Sigma HPA001893, and rabbit polyclonal anti-SIX1 Cell Signaling Technology D4A8K were validated by shRNA-mediated knockdown in house. Rabbit polyclonal anti-FLI1 Abcam ab15289 and ab133485 validated by shRNA-mediated or siRNA-mediated knockdown in house. Mouse monoclonal anti- β-Actin Sigma A5316 validated for use in Western blotting of human protein by the manufacturer (https://www.sigmaaldrich.com/US/en/product/sigma/a5316). Mouse monoclonal anti-HDAC1 Santa Cruz Biotechnology Sc-81598 validated for use in Western blotting of human protein by the manufacturer (https://www.scbt.com/p/hdac1-antibody-10e2?gclid=Cj0KCQjwnP-ZBhDiARIsAH3FSRdLo2orY5kOjoMwmfaivctZ0EdSHcSXQmPleIe-l8UgX98_AkhHZkUaArFKEALw_wcB). Rabbit polyclonal anti-Integrin B1 Cell Signaling Technology 4706S validated for Western blotting on human protein by the manufacturer (https://www.cellsignal.com/products/primary-antibodies/integrin-b1-antibody/4706). Rabbit monoclonal anti-integrin A1 Cell Signaling

Technology 15574S validated for Western blotting of human protein by the manufacturer (https://www.cellsignal.com/products/primary-antibodies/integrin-a1-cd49a-e9k2j-xp-rabbit-mab/15574). Rabbit monoclonal anti-integrin A2 ThermoFisher Scientific MA5-32306 validated for Western blotting on human protein by the manufacturer (https://www.thermofisher.com/antibody/product/CD49b-Integrin-alpha-2-Antibody-clone-SN0752-Recombinant-Monoclonal/MA5-32306). Mouse monoclonal anti-integrin A4 Santa Cruz Biotechnology Sc-365209 was validated for Western blotting of human protein by the manufacturer (https://www.scbt.com/p/integrin-alpha4-antibody-a-7). Goat polyclonal anti-integrin A5 R&D Systems AF1864 was validated for Western blotting of human protein by the manufacturer (https://www.rndsystems.com/products/human-integrin-alpha5-cd49e-antibody_af1864https://www.rndsystems.com/products/human-integrin-alpha5-cd49e-antibody_af1864). Rabbit polyclonal anti-integrin A6 Cell Signaling Technology 3750S was validated for Western blotting of human protein by the manufacturer (https://www.cellsignal.com/products/primary-antibodies/integrin-a6-antibody/3750). Mouse monoclonal anti-FAK Invitrogen AHO1272 was validated for Western blotting of human protein by the manufacturer (https://www.thermofisher.com/antibody/product/FAK-Antibody-clone-34Q36-Monoclonal/AHO1272). Rabbit monoclonal anti-p-FAK (Y397) Abcam ab81298 was validated for Western blotting of human protein by the manufacturer (https://www.abcam.com/products/primary-antibodies/fak-phospho-y397-antibody-ep2160y-ab81298.html). Mouse monoclonal anti-SRC Invitrogen AHO1152 was validated for Western blotting of human protein by the manufacturer (https://www.thermofisher.com/antibody/product/SRC-Antibody-clone-184Q20-Monoclonal/AHO1152). Rabbit monoclonal anti-p-SRC (Y416) Cell Signaling Technology 6943S was validated for Western blotting of human protein by the manufacturer (https://www.cellsignal.com/products/primary-antibodies/phospho-src-family-tyr416-d49g4-rabbit-mab/6943). Rabbit polyclonal anti-NaKATPase Cell Signaling Technology 3010s was validated for Western blotting of human protein by the manufacturer (https://www.cellsignal.com/products/primary-antibodies/na-k-atpase-antibody/3010).

Eukaryotic cell lines

Policy information about cell lines and Sex and Gender in Research

Cell line source(s)

A673 (RRID:CVCL_0080) - Sex: Female - Species: Homo sapiens. Purchased from ATCC.
EWS-502 (RRID:CVCL_S740) - Sex: Unspecified - Species: Homo sapiens. Acquired as a generous gift from the laboratory of Dr. Stephen Lessnick (The Ohio State University).
TC-71 (RRID:CVCL_2213) - Sex: Male - Species: Homo sapiens. Acquired as a generous gift from the laboratory of Dr. Stephen Lessnick (The Ohio State University). Available via the Childhood Cancer Repository cell bank.
SK-ES-1 (RRID:CVCL_0627) - Sex: Male - Species: Homo sapiens. Purchased from ATCC.
SK-N-MC (RRID:CVCL_0530) - Sex: Female - Species: Homo sapiens. Purchased from ATCC.
Human Mesenchymal Stem Cells (hMSCs) were purchased from Lonza Biosciences (PT-2501) - Sex: Not specified - Species: Homo sapiens
A673 doxycycline-inducible EWS-FLI1 knockdown cells acquired as a generous gift from the laboratory of Dr. Olivier Delattre (Institut Curie).

Authentication

All cell lines used were authenticated in 2015 (Niemeyer, B. F. et al. 2015). A673 and EWS-502 were again STR-profiled by the CU Cell Technologies Shared Resource in 2019 to confirm authentication. A673 and EWS-502 SCR and SIX1 KD cells were also STR profiled in 2023.

Mycoplasma contamination

All cell lines were tested every 3-6 months in culture for mycoplasma contamination and were confirmed negative throughout study.

Commonly misidentified lines
(See ICLAC register)

SK-N-MC is listed as a commonly misidentified cell line. As per the ICLAC register, SK-N-MC is commonly misidentified as a human neuroblastoma cell lines, however its correct identification is Ewing's sarcoma, which is what it is used as a model of in this context.

Animals and other research organisms

Policy information about studies involving animals; ARRIVE guidelines recommended for reporting animal research, and Sex and Gender in Research

Laboratory animals

For in vivo studies, three- to twelve-week-old NOG/SCIDg (NSG) male mice (Mus musculus) were used.
Housing conditions:
Light cycle: 14h light: 10h dark
Temperature: 72°F +/- 2°F
Humidity: 40% +/- 10%
Water: Hyperchlorinated (2-5 ppm) Reverse Osmosis delivered via automatic watering
Food: Teklad (Envigo) diets.
Rodents - Standard diet (2920X). Breeder diet (2919) both irradiated.

Wild animals

This study did not involve wild animals

Reporting on sex

Male mice were prioritized in the in vivo tail vein study design, as males are disproportionately represented in Ewing sarcoma incidence (over 60% of cases) and have significantly worse outcomes as compared to females (Jawad, M. U. et al 2009). Both male and female mice were used for the A673 subcutaneous tumor model.

Field-collected samples

This study did not involve samples collected from the field.

Ethics oversight

The work compiled in this paper complies with Institutional Biosafety Committee (Protocol #1140) regulations. All animal studies were performed according to protocols reviewed and approved by the Institutional Animal Care and Use Committee (IACUC) at the University of Colorado AMC (Protocol #00089).

Note that full information on the approval of the study protocol must also be provided in the manuscript.
